# ESGI: efficient splitting of generic indices in single-cell sequencing data

**DOI:** 10.1093/bib/bbag631

**Published:** 2026-11-15

**Authors:** Tim Stohn, Nadine D van de Brug, Anastasia Theodosiadou, Bram Thijssen, Katarzyna Jastrzebski, Lodewyk F A Wessels, Evert Bosdriesz

**Affiliations:** Computer Science Department, Vrije Universiteit Amsterdam, De Boelelaan 1111, 1081 HV Amsterdam, The Netherlands; Division of Molecular Carcinogenesis, The Oncode Institute, The Netherlands Cancer Institute, Plesmanlaan 121, 1066 CX, Amsterdam, The Netherlands; Computer Science Department, Vrije Universiteit Amsterdam, De Boelelaan 1111, 1081 HV Amsterdam, The Netherlands; Division of Molecular Carcinogenesis, The Oncode Institute, The Netherlands Cancer Institute, Plesmanlaan 121, 1066 CX, Amsterdam, The Netherlands; Computer Science Department, Vrije Universiteit Amsterdam, De Boelelaan 1111, 1081 HV Amsterdam, The Netherlands; Division of Molecular Carcinogenesis, The Oncode Institute, The Netherlands Cancer Institute, Plesmanlaan 121, 1066 CX, Amsterdam, The Netherlands; Division of Molecular Carcinogenesis, The Oncode Institute, The Netherlands Cancer Institute, Plesmanlaan 121, 1066 CX, Amsterdam, The Netherlands; Division of Molecular Carcinogenesis, The Oncode Institute, The Netherlands Cancer Institute, Plesmanlaan 121, 1066 CX, Amsterdam, The Netherlands; Faculty of EEMCS, Delft University of Technology, Van Mourik Broekmanweg 6, 2628 XE, Delft, The Netherlands; Computer Science Department, Vrije Universiteit Amsterdam, De Boelelaan 1111, 1081 HV Amsterdam, The Netherlands

**Keywords:** demultiplexing, UMI-based quantification, single-cell sequencing, preprocessing, indel-aware matching, barcodes

## Abstract

Single-cell sequencing technologies increasingly rely on complex nucleotide barcoding schemes to encode cellular identities, experimental conditions, and molecular modalities within a single experiment. While demultiplexing, alignment, and unique molecular identifier (UMI)-based quantification form the core preprocessing steps that transform raw sequencing reads into analyzable single-cell data, existing pipelines are often tightly coupled to specific experimental designs and typically assume fixed barcode positions and substitution-only error models. As a result, many assays employing combinatorial, variable-length, or multimodal barcoding designs require custom, hard-coded preprocessing solutions that are difficult to generalize and maintain. Here, we present efficient splitting of generic indices (ESGI), a flexible framework for demultiplexing and processing single-cell sequencing data with arbitrary barcode architectures. ESGI operates directly on raw FASTQ files using a generic barcode pattern specification, supports barcode matching with insertions and deletions via Levenshtein distance, accommodates variable-length barcodes, and provides detailed quality metrics for barcode assignment. ESGI optionally integrates genome alignment via STAR and performs feature quantification and UMI collapsing to generate cell-by-feature count matrices. ESGI is well documented and readily applicable to novel single-cell experiments. We demonstrate the versatility of ESGI across six datasets spanning four distinct single-cell technologies, including combinatorial indexing–based transcriptomic and multimodal assays, feature barcode-based protein measurements, and spatial barcoding data. Across these applications, ESGI robustly demultiplexes complex barcode designs that are not natively supported by existing pipelines, while producing results comparable with established workflows where applicable. ESGI provides a general solution for processing a wide range of barcoded single-cell sequencing data, enabling rapid adoption and analysis of emerging experimental designs.

## Introduction

Single-cell sequencing has evolved from scRNA-seq assays to a rapidly growing array of sequencing-based technologies that measure diverse molecular modalities at single-cell resolution. Central to many of these technologies is the use of nucleotide barcodes, which are employed to uniquely label individual cells, spatial coordinates [1], experimental conditions, modalities like protein [2–4], transcriptome [5, 6], chromatin accessibility [7], guides in CRISPR-based single-cell perturbations [8], combinations of them [9–11] and much more. The process of identifying the barcodes present in a sequencing read and thereby assigning the reads to their respective categories, such as individual cell identities or experimental conditions, is commonly referred to as demultiplexing. The measured features may comprise nucleotide sequences, such as DNA or RNA, which must be aligned to a reference genome prior to quantification, or feature barcodes, such as nucleotide-tagged antibodies whose sequences provide direct readouts of, e.g. the abundance of the proteins bound by the antibodies. Single-cell identities are generally encoded either by a single barcode [11, 12] or by a combination of multiple barcodes [3, 6]. Following demultiplexing and mapping to the reference genome, the measured modalities are quantified for all cells. This is often done using unique molecular identifiers (UMIs) to identify PCR duplicates and obtain accurate molecule-level abundance estimates. Together, demultiplexing, mapping of genomic sequences and counting single-cell features (UMI collapsing) constitute the core processing steps that transform raw sequencing reads into cell-by-feature count matrices for downstream single-cell analysis. As barcoding schemes have increased in number and complexity, demultiplexing has become an increasingly technically challenging preprocessing step.

Several tools exist to process barcoding-based single-cell data, but most are designed around specific experimental platforms and offer limited flexibility for novel barcoding schemes. Commercial platforms provide dedicated pipelines that are robust and well-maintained, e.g. Cell Ranger for 10*× * Genomics, but these are tightly coupled to their respective assay designs. STARsolo is a more flexible tool that performs integrated alignment, barcode processing, and gene quantification for a range of single-cell assays, and supports flexible barcode positioning within a single read, but cannot handle barcode segments spanning both reads of a paired-end library [13]. zUMIs supports user-defined barcoding schemes for single-cell RNA-seq data but is not designed for feature-barcodes or multimodal assays [14]. Alevin and its successor alevin-fry provide efficient, alignment-free quantification with flexible specification of barcode and UMI positions [15, 16]. kallisto and bustools enable rapid, pseudoalignment-based processing of single-cell data through a modular framework that accommodates arbitrary and combinatorial barcode schemes [17], and the kITE workflow extends this framework to feature barcode assays [18]. SDRRanger was developed for joint single-cell RNA and DNA sequencing with support for flexible barcode positioning [19]. Across these tools, a common limitation is that barcodes are extracted at fixed, predefined positions within the read, meaning the starting position of each barcode element is assumed rather than inferred from the data.

A second, related limitation concerns the correction of mismatches in barcodes. Barcode sequence similarity is commonly measured using either Hamming or Levenshtein distance. Hamming distance counts positional mismatches between equal-length sequences, allowing only for substitutions, whereas Levenshtein distance additionally accounts for insertions and deletions (indels). Most tools rely exclusively on Hamming distance for barcode matching, and therefore only account for nucleotide substitutions and not indels during barcode mapping. This distinction matters because indels occurring early in a read shift the expected positions of all downstream barcode elements, causing them to be misassigned even when the barcodes themselves are error-free. Such positional shifts are particularly consequential in combinatorial barcoding schemes, where multiple barcode elements are concatenated in a single read. These errors in barcodes can arise from various sources, such as errors in the synthesis of barcodes, errors during library preparation due to PCR amplification errors, or sequencing errors. Library preparation errors have been reported to occur at negligible rates below $10^{-5}$ per base for high-fidelity DNA polymerases [20]. Illumina short-read sequencing exhibits per-base error rates on the order of $10^{-3}$–$10^{-2}$, with sequencing errors dominated by single-base substitutions rather than insertions or deletions [21, 22]. However, deletions are reported to be the most dominant errors in barcode synthesis [23], and Hawkins *et al*. explicitly show that deletions dominate synthesis errors in their barcoding experiments [24]. To address this, they recently developed an indel-correcting method called FREEbarcodes and implemented it in their demultiplexing pipeline SDRRanger [19], but indel-aware demultiplexing remains unavailable in most tools. Overall, current demultiplexing tools are frequently limited by fixed barcode extraction and substitution-only error correction. This prevents their application to complex barcoding patterns that demand both mapping flexibility and indel-aware barcode handling.

To allow for flexible pattern matching *and* handle indels in barcodes, we developed efficient splitting of generic indices (ESGI), a flexible demultiplexing and processing framework designed to support complex single-cell barcoding schemes. ESGI takes as input raw FASTQ files together with a flexible barcode pattern specification that describes the order and allowed sequences of barcodes within each read. Based on this specification, ESGI performs barcode demultiplexing, optionally aligns genomic read sequences to a reference genome using STAR, and subsequently counts single-cell features and collapses UMIs to generate a final single-cell feature matrix. In contrast to existing tools, ESGI supports barcode matching with insertions and deletions via Levenshtein distance, can map variable-length barcodes (staggers) [2], and reports detailed quality metrics that provide insight into barcode assignment and mapping performance. As single-cell technologies continue to evolve toward increasingly complex barcoding patterns and multimodal designs, ESGI offers a flexible and easy-to-use solution for emerging methods that lack dedicated bioinformatics pipelines, while providing additional statistical information that may inform further optimization of experimental protocols. ESGI is extensively documented, and we provide vignettes to run ESGI on diverse single-cell technologies.

To demonstrate its versatility and performance, we applied ESGI to six datasets of four different studies representing four distinct barcoding-based single-cell sequencing technologies. The datasets cover various barcoding technologies as well as RNA and protein measurements. The technologies include combinatorial indexing data profiling both RNA and protein abundance, a 10*× *-based technology measuring RNA and protein simultaneously and a novel spatial barcoding technology. First, to demonstrate the advantages of flexible and indel-aware debarcoding we applied ESGI to two combinatorial indexing datasets: data generated with SIGNAL-seq, a recently developed single-cell method employing combinatorial barcoding to jointly profile RNA and protein modalities [6], and a dataset generated using the SPLiT-seq technology [25], representing one of the original combinatorial barcoding approaches for single-cell transcriptomics. Next, we applied ESGI to Phospho-seq, which simultaneously quantifies RNA and protein abundances using 10*× * Genomics single-cell barcoding in combination with custom nucleotide-tagged antibodies [26]. Finally, we evaluated ESGI on xDBiT data, a parallelized spatial barcoding technology that employs a custom barcode design to encode spatial locations together with sample indices to process nine samples in parallel [1]. While the original xDBiT workflow relies on a custom pipeline built upon tools developed for Drop-seq data [27], we show that ESGI can robustly process this dataset with its flexible framework.

## Materials and methods

### Overview of ESGI

ESGI enables flexible demultiplexing and counting of barcoding-based single-cell data, such as multi-modal, combinatorial barcoding, or spatial data (Fig. 1a). Input to ESGI are the FASTQ files that have to be processed together with a user-provided barcode pattern (Fig. 1b). The predefined generic input pattern allows for various elements at different positions in the FASTQ reads, so ESGI can easily be applied to novel sequencing technologies with new barcoding combinations. This input pattern consists of several elements that together give a generic description of the expected nucleotide sequence in the FASTQ reads. These elements can be barcode elements where a sequence from a whitelist of potential barcodes can occur, constant elements, UMIs, or genomic sequences like DNA/RNA. Barcode elements in the pattern can have many possible barcode sequences that can, e.g. encode single-cell indices, feature indices, or hash tags. Constant elements are predefined nucleotide sequences that, e.g. link other elements in the pattern. Additionally, the user can set the number of allowed mismatches for every element individually or decide if a barcode should be mapped by Levenshtein distance, which allows for both substitutions and indels, or only by Hamming distance, which allows for substitutions only, but can be more efficient in some instances.

**Figure 1.**
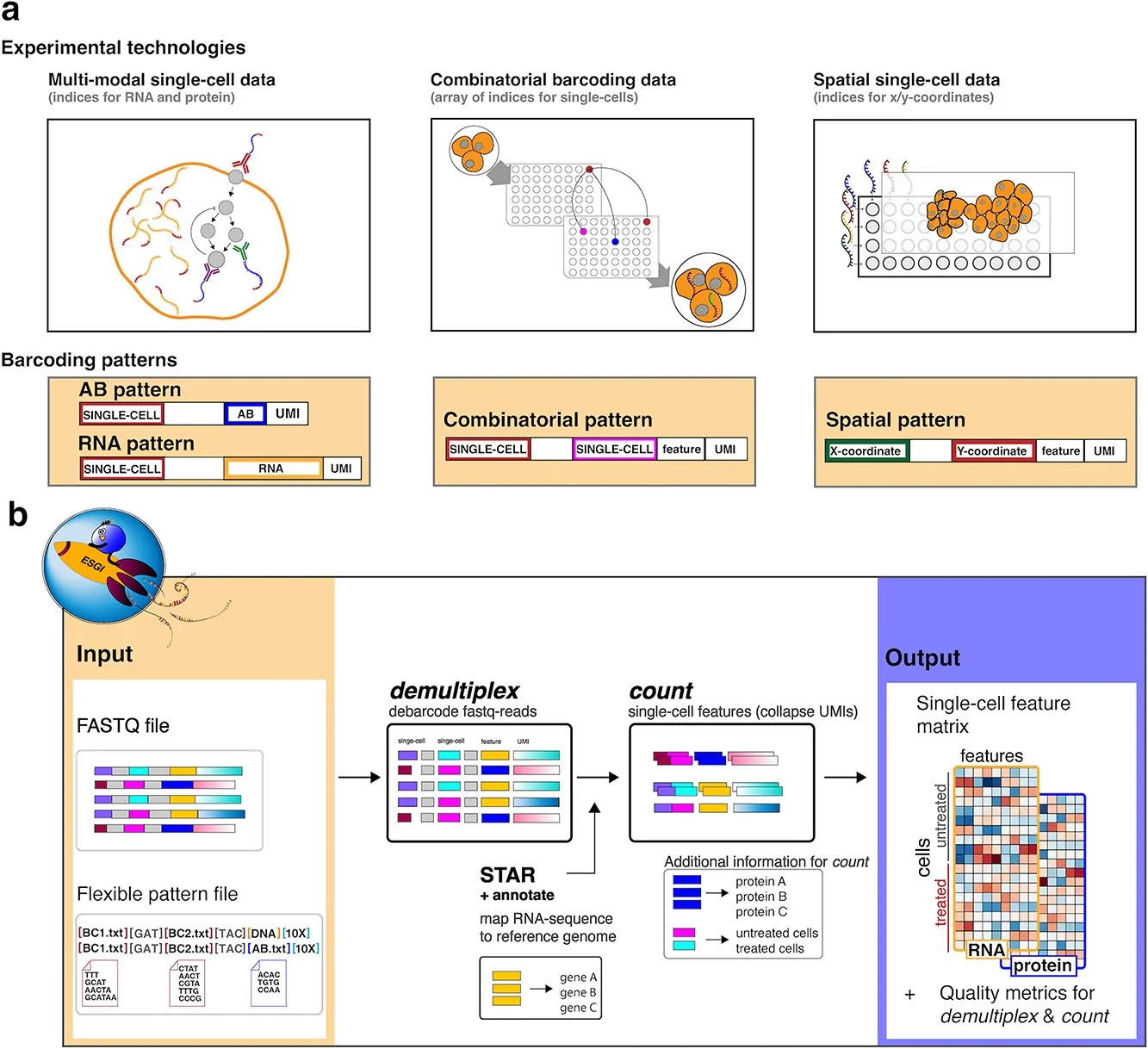
Workflow of ESGI. (a) ESGI can demultiplex various data types: multimodal data, with an RNA-sequence and an antibody-attached (AB) feature barcode encoding the protein modality, combinatorial barcoding data, where the combination of barcodes encodes for individual cells, or spatial data with barcodes encoding coordinates. (b) Input to ESGI are the raw FASTQ files and a flexible pattern which describes the barcoding scheme as a list of barcode elements. ESGI then maps the patterns to the FASTQ reads. ESGI is comprised of two main tools: demultiplex and count. demultiplex maps the expected barcodes in the FASTQ file and count counts the number of reads for single-cell features by collapsing UMIs. If the data contain a ‘DNA’ element, ESGI can call STAR and create a genome annotation that is added to the output of demultiplex with the tool annotate before calling count.

ESGI can be split into two separate steps: demultiplexing the sequence pattern and counting single-cell features. ESGI can be run as a wrapper of these two tools, but it can also be split into separate calls of the internal tools demultiplex and count for more control. During the demultiplexing step the FASTQ reads are split into the elements as defined in the input pattern. Elements are corrected if they are within the mismatch limit. If genomic sequences are present, ESGI can call STAR to map these sequences to a reference genome and add the STAR annotation to the output of demultiplex. The counting step collapses UMIs, generates a single-cell feature matrix, and assigns additional barcoded attributes, like treatment conditions, if these are present in the barcode pattern. In addition to the final data matrix ESGI generates quality metrics for the demultiplexing and counting step.

### Demultiplexing

ESGI demultiplexes barcoding-based single-cell data by mapping the input reads to a predefined generic input pattern. In this pattern, every barcode element is enclosed within square brackets and can consist of constant linker sequences, barcode elements, random elements, or a genomic sequence. Constant linker elements are described by the exact nucleotide sequence. Barcode elements, which can, e.g. be used to identify individual cells, are specified in whitelist files (e.g. ‘BC1.txt’) that contain all valid barcode sequences for this barcode element. ‘DNA’ denotes genomic or transcriptomic sequence content to be aligned to a reference genome, and random sequences of length *L*, such as UMIs, are described as ‘*LX*’ (Fig. 1b). ESGI maps individual pattern elements sequentially along the read allowing for substitutions and indels. To do so, ESGI precomputes all possible sequences with a Hamming distance of one for each constant element and all barcodes of the barcode elements. ESGI starts with the first pattern element, extracts the expected sequence length from the input read, and checks whether the extracted sequence matches any of the expected barcodes or the precomputed sequences with a mismatch. By default, ESGI also allows for insertions and deletions. To that end, ESGI also precomputes all possible sequences with one insertion or deletion (unless the user explicitly requests Hamming distance only). The start of the following pattern element is defined by the end position of the currently mapped pattern element. In contrast to extracting pattern elements from fixed positions, this approach prevents shifts in the mapping frame that would otherwise propagate to downstream barcodes (Fig. 2a).

**Figure 2.**
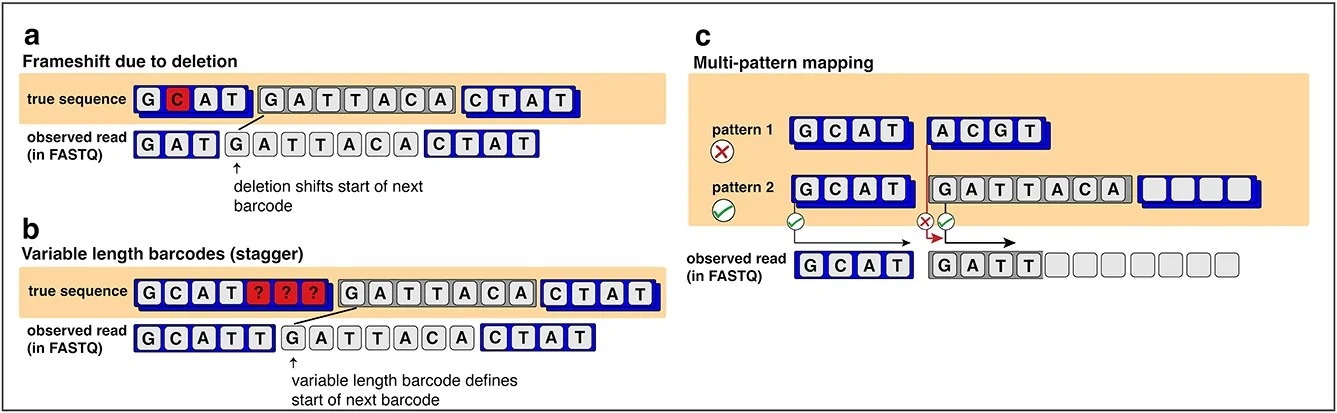
ESGI’s flexible framework enables effective handling of novel and previously unaddressed scenarios. (a) ESGI maps barcodes allowing for substitutions and indels. Indels can lead to a shift in the mapping-frame which results in new starting positions for subsequent barcodes. (b) Variable length barcodes can make it impossible to ‘cut’ barcodes at fixed positions in the read. By mapping barcodes sequentially ESGI can map barcodes of variable length and starts at the new offset with the subsequent barcode. Therefore, the starting position of a barcode depends on the mapping of the previous barcode. (c) ESGI can demultiplex several patterns at once in cases where a FASTQ file contains several modalities that were captured and sequenced together.

If a pattern contains barcode elements and more than one mismatch is allowed, ESGI aligns potential barcode sequences to the input reads. For barcode elements ESGI uses positional k-mers to reduce the search space for possible barcodes at this position. To do so, ESGI calculates all positional k-mers of the whitelist barcodes and before mapping filters out barcodes that do not share a minimum number of positional k-mers with the extracted sequence (Fig. S2). The remaining barcodes are then aligned to the input sequence using a bit-parallel algorithm as implemented in the Edlib library [28]. ESGI aligns barcodes to the input reads using a semi-global alignment strategy. This approach permits unpenalized terminal deletions in the extracted sequence that is mapped to the barcode, and thereby prevents excess bases at the end of the extracted sequence that do not map to the barcode from being counted (for instance when a deletion in the barcode sequence shortens the effective barcode length in the extracted sequence) (Fig. S3). As a result mismatches are not artificially inflated, in contrast to a pure Levenshtein distance computed between equal-length sequences, similar to the idea of FREEbarcodes [24]. To further improve the running time, ESGI precomputes all pairwise distances between barcodes of a barcode element if the number of possible barcodes is below 500. During mapping, these distances are used to terminate alignments early if all remaining unchecked barcodes must exceed the current best alignment score. The precomputed distances yield a matrix of shortest possible edit distances between all theoretically attainable barcodes. During the alignment of barcodes to the FASTQ reads, this information allows ESGI to terminate the search early once the closest possible match is identified. Concretely, suppose a candidate barcode *b* aligns to a sequence *s* with edit distance *d(b, s)*. Let $d_{\min }(b, b^{\prime})$ denote the minimum edit distance between *b* and any other barcode $b^{\prime} \neq b$. If $d(b,s) < d_{\min }(b,b^{\prime}) - d(b,s)$, then no alternative barcode can achieve a smaller alignment distance to the sequence. Hence, *b* is guaranteed to be the optimal match, and ESGI can safely terminate the alignment procedure. ESGI discards reads if the detected barcode at a barcode element is ambiguous and more than one barcode would map with a minimal edit distance.

Barcoding-based single-cell data can contain barcodes that are of variable length. These are often introduced by staggers, which result in offsets in barcodes and are useful to increase base diversity and thereby improve cluster detection and base calling during sequencing [29]. However, variable length barcodes cannot be ‘cut out’ at fixed positions in the sequence. Therefore, ESGI tries to map all possible barcodes with variable lengths, reports the result if one single barcode fits best with minimal edit-distance and continues mapping the subsequent pattern element at the end position of this sequence (Fig. 2b).

ESGI can simultaneously map multiple patterns to a FASTQ file, enabling demultiplexing when sequencing libraries have not been separated prior to sequencing (Fig. 2c). ESGI therefore tries to map all possible patterns, and if one pattern matches with minimal edit-distance reports this pattern. This capability also allows the specification of more flexible barcode schemes, e.g. when the validity of one pattern element depends on the identity of another element and only specific barcode combinations are permitted. By supporting multiple patterns, ESGI simplifies the demultiplexing process and makes conditional and hierarchical barcode designs possible (Supplementary Information 1.1).

### Calling STAR

ESGI calls STAR (version *≥ * 2.7.9) as an external aligner to obtain genomic coordinates and gene annotations. demultiplex separates the genomic region of the input reads from the remaining pattern elements and writes the genomic reads into a FASTQ file and the remaining pattern elements into a .tsv file. STAR is invoked in non-solo mode to produce genomic BAM files containing GX and GN tags for the FASTQ file containing only the genomic reads without barcoding information, where GX denotes the Ensembl gene identifier (ENSG) and GN the corresponding gene symbol. ESGI then extracts these tags via an internal annotation step and associates each read with its gene identity. The resulting gene annotations are merged with the demultiplexed pattern elements (.tsv file) to generate demultiplexed reads with gene-annotations, which can be used as input to count.

### Feature counting

The final step of ESGI is counting single-cell feature occurrences with the tool count. Input for this step is either the output of demultiplex directly or the STAR-annotated output of demultiplex if genomic sequences (as in scRNA-seq) are present. In this step UMIs are collapsed and counted to generate the final single-cell feature matrix. First, reads are sorted by single-cell and feature barcodes and UMIs are only collapsed if they come from the same feature in the same cell. Therefore, UMIs are initially counted based on exact matches and subsequently ordered by their count. For the same single-cell feature combination identical UMIs are collapsed into a single count for that feature in that cell. Sequencing errors can result in UMIs originally arising from the same molecule to differ in the sequenced read. To account for this, we employ a strategy similar to alevin [15]. Specifically, starting with the most abundant UMI, we compare it to all UMIs from the same feature in the same single-cell with equal or fewer counts. If the distance between two UMIs is below a threshold, the less abundant UMI can be collapsed into the more abundant one (by default allowing a Hamming distance of one). UMIs are only collapsed if the less abundant UMI has <20% of the counts of the more abundant UMI. For more detailed information, see Supplementary Information 1.3.

### Simulation of reads from barcoded single-cell data

To benchmark ESGI against a known ground truth, we simulated single-cell sequencing reads following a combinatorial split-pool barcoding design with feature barcodes similar to SIGNAL-seq [6]. Each read was assembled with the following structure: $\mathrm{[BC1]}\;\mathrm{[Linker1]}\;\textrm{[UMI]}\;\mathrm{[BC2]}\;\mathrm{[Linker2]}\;\textrm{[BC}_{\textrm{feature}}\textrm{]}$. Two 8 bp cell barcodes (BC1 and BC2) jointly define the combinatorial cell identity, a 10 bp UMI enables read de-duplication, and an 8 bp feature barcode (BC$_{\textrm{feature}}$) encodes the captured protein-signal. The two barcodes were separated by fixed linker sequences of 22 bp (Linker1) and 30 bp (Linker2), giving a total read length of 86 bp. For the linker sequences we used the same sequences that were used as linker elements in the SIGNAL-seq technology.

Cell and feature barcodes were generated as random sequences of the four possible bases, with a minimum pairwise Hamming distance of 3 within each set to ensure recoverability after the introduction of sequencing errors. By default we generated a pool of 50 cell barcodes (drawn for both BC1 and BC2) and 20 feature barcodes. For each read, BC1, BC2, and the feature barcode were drawn uniformly at random from their respective pools, and a fresh 10 bp UMI was generated by uniform random sampling of nucleotides. UMIs were drawn at random from the full 10 bp sequence space and assigned independently per read. We did not explicitly model UMI saturation or PCR duplication; consequently, with $1 \times 10^{6}$ reads over a $4^{10}$ UMI space, the probability that two distinct molecules share a UMI within a cell–feature combination is negligible. Count-level comparisons should therefore be interpreted as reflecting barcode and feature recovery accuracy rather than the performance of any particular UMI-collapsing strategy.

To emulate sequencing errors, each read was assigned between 1 and 3 errors (drawn uniformly), with each error independently chosen to be a substitution, insertion, or deletion with equal probability and placed at a uniformly random position along the read. Substitutions replaced a base with one of the three alternative nucleotides; insertions added a random nucleotide; and deletions removed a base, such that indels altered the read length and shifted the positions of all downstream barcode elements relative to the error-free layout. For every read we recorded the true BC1, BC2, feature barcode, UMI, and the error-free coordinates of each barcode element as ground truth. We simulated $1\times 10^{6}$ reads in total.

Three tools were evaluated: ESGI (run in two configurations—wildcard linkers and explicit linker matching with up to 3 mismatches per linker), kITE (kallisto/bustools v0.51.1 using a Hamming-distance mismatch index built with kb ref –workflow kite), and alevin (salmon alevin with sketch-based mapping, followed by alevin-fry for barcode correction and quantification with parsimony-EM). When run with wildcard linkers ESGI skips the linker sequence and continues barcode mapping at the end of the linker sequence.

Accuracy was assessed at two levels. Before UMI collapsing (read level), each individual read was traced back to its ground-truth identity via the read name: ESGI reports read names directly in its per-read TSV output; for kITE, kallisto bus was re-run with the –num flag, which writes the 0-based read index into the BUS flag column, and the raw BUS file was then sorted, barcode-corrected, and re-sorted before conversion to text with bustools text -f, yielding one row per aligned read with its assigned barcode, UMI, equivalence class, and read index. Each read was classified as TP (cell barcode and feature both correct), FP (one or both wrong), or FN (not recovered). After UMI collapsing (count level), the final count matrices from each tool were compared to the expected per-(cell, feature) UMI counts derived from the ground truth; count-level TP (recovered counts = min(tool, expected)), FP (excess counts), and FN (missing counts) were computed, alongside Pearson correlation and mean absolute error. Alevin processes reads through an internal mapping and collation pipeline (salmon alevin  *→ *  alevin-fry generate-permit-list  *→ *  collate  *→ *  quant) that does not expose per-read barcode or feature assignments; only the final aggregated count matrix is accessible. It was therefore included in the post-UMI count-level comparison only, where the number of mapped reads from the alevin-fry $meta\_{info.json}$ was used as a proxy for recall.

### Benchmarking ESGI on SIGNAL-seq data

To demonstrate the applicability of ESGI to novel and highly multiplexed barcoding schemes, we applied ESGI to a dataset generated using SIGNAL-seq technology, which labels individual cells through three rounds of combinatorial barcoding and jointly profiles RNA and protein modalities using feature barcodes [6]. We used the datasets of HeLa spheroid cells which are publicly available from GEO (accession GSE256405), which were downloaded from the Sequence Read Archive (SRA run SRR28056729 for the RNA modality and SRA run SRR28056728 for the protein modality).

The authors of SIGNAL-seq processed the protein modality with kITE [18]. Since kITE only maps barcodes at the expected positions we ran ESGI without explicitly mapping the constant linker regions between barcode elements unless stated differently. As kITE permits one substitution per barcode element, we applied the same mismatch tolerance in ESGI by allowing one mismatch in each barcode element. kITE does not allow for mismatches in the extracted UMI. Therefore, we also ran ESGI with zero mismatches in the UMI during counting of the single-cell feature barcodes with count. The authors of SIGNAL-seq processed the RNA modality with zUMIs [14]. zUMIs concatenates the three barcode sequences before error correction, and we ran it with three substitutions for the concatenated sequence. Similarly, we allowed 1 mismatch in every barcode element with ESGI. For the RNA modality, both zUMIs and ESGI were executed with UMI error correction permitting a single nucleotide substitution in the UMI sequence. Since ESGI supports only counting of exonic reads we ran zUMIs without counting intronic reads. The data contains reads from oligo-dT and random hexamer reverse transcription primers and both reads have different barcodes. zUMIs was run to assign both reads to the same cell and we therefore also ran ESGI by supplying a barcode-mapping file, which combines barcodes and assigns reads from both barcodes to the same cell. To compare the results obtained by ESGI to those obtained by kITE/zUMIs, we computed per-cell correlations between feature counts of the compared tools across all measured features. For kITE we took the unfiltered counts and removed cells for which correlations could not be computed (due to no detected features or zero variance). For the comparison of RNA counts we separately prefiltered the output of ESGI and zUMIs to include only cells for which the tools detected >100 counts, calculated the per-cell correlations and also excluded cells for which correlations could not be computed.

To enable systematic comparison of clustering results across tools, we aligned cluster labels between ESGI and each reference tool using a greedy one-to-one matching procedure. For each pair of tools, we computed a contingency table counting the number of shared cells between every pair of clusters across the two tools. Clusters were then matched iteratively: at each step, the pair with the largest cell overlap was assigned a shared numeric label, and both clusters were removed from the table before proceeding to the next iteration. This continued until no further matches could be made. Matched clusters were assigned identical labels and colors across both UMAP panels to facilitate visual comparison. Clusters that could not be matched to any cluster in the other tool were retained and labeled as tool-specific. All downstream concordance metrics—including within-cluster per-feature Pearson correlations—were computed on the set of cells shared between both tools within each aligned cluster pair.

### Benchmarking ESGI on Phospho-seq data

Many single-cell experiments are run with 10*× * or are a combination of custom protocols that are combined with 10*× *. While 10*× * data can be easily demultiplexed and processed with Cell Ranger, custom protocols might need adjustment. Therefore, to show ESGI’s compatibility with 10*× * and its ability to build upon their barcoding scheme, we processed scRNA-seq and protein quantification data of the Phospho-seq technology with ESGI [26]. This dataset contains a single 10*× * single-cell barcode, while the protein modality is encoded using feature barcodes. We downloaded two datasets of the Phospho-seq-Multi experiment for the RNA and protein modality. The data are publicly available from GEO (accession GSE285561) and were downloaded from the Sequence Read Archive (SRA run SRR31955816 for the RNA modality and SRR31955815 for the protein modality). The authors processed the protein modality with alevin and the RNA modality with 10*× * Genomics Cell Ranger. Therefore, we processed both modalities in a similar fashion with ESGI and compared the results with the results of alevin for the protein and with Cell Ranger for the RNA modality. For the comparison of ESGI with Cell Ranger, we generated a GRCh38 reference using Cell Ranger (v9.0.1) with Gencode v43. The resulting Cell Ranger–processed genome FASTA and gene annotation were reused to construct a separate STAR (v2.7.10b) genome index for the ESGI pipeline, ensuring identical reference sequences and gene models across analyses. We excluded intronic regions for the Cell Ranger run for comparability with ESGI. We then ran ESGI allowing for one mismatch in the single-cell barcode and collapsed UMIs with a Hamming distance of one. To calculate correlations between the results obtained by the methods, we used the raw unfiltered output of Cell Ranger and excluded cells for which we could not calculate a correlation due to no detected features in one of the tools or zero variance. Alevin allows for one mismatch (Hamming distance) in the single-cell and feature barcode and we ran ESGI as well with one mismatch. Similar to alevin we ran ESGI allowing for one substitution in UMIs during UMI collapsing. To calculate correlations for the results of both tools, we filtered single cells that had at least five counts across all measured proteins and we excluded cells that were not detected in one technology or had zero variance in the protein counts.

### Benchmarking ESGI on xDBiT data

We also applied ESGI to spatial data. To showcase the ease of application to novel sequencing technologies we applied ESGI to a new multiplexed spatial technology, which barcodes spatial coordinates as well as sample identities to process nine tissue samples in parallel [1]. The data contain three barcode elements—one for each of the x- and y-coordinates, as well as a third barcode to distinguish between nine tissue samples. The three elements are connected by two 30 nucleotide long constant linker elements. We analyzed the first replicate of the mouse Cerebellum tissue, which is publicly available from GEO (accession GSE207843) and was downloaded from the Sequence Read Archive (SRA run SRX16111595). We compared the output of ESGI to the output of the xDBiT toolbox using the precomputed single-cell count matrix provided by the authors via their GitHub repository (xDBiT_toolbox). We ran ESGI allowing for one mismatch in the barcode elements and when running ESGI in the mode where we also explicitly map the constant elements, we allowed 10 mismatches in both linker elements. We collapsed UMIs allowing for one substitution in the UMI sequence. For the correlation analysis, we excluded cells for which no correlation could be computed due to missing counts or zero variance.

### Analysis of SPLiT-seq data

SPLiT-seq (Split Pool Ligation-based Transcriptome sequencing) is a combinatorial indexing method for single-cell RNA sequencing that labels transcripts through successive rounds of split–pool barcoding [25]. In each round, cells are redistributed across wells and tagged with a new barcode, and the combination of barcodes uniquely identifies the cell of origin after sequencing. To count the number of mismatches in the SPLiT-seq data we ran demultiplex on a small mouse brain dataset of 100 nuclei that was generated with the SPLiT-seq technology and also used in a benchmarking study of pipelines for SPLiT-seq data [30]. The data contain three barcode elements that are eight nucleotides long and are connected by two 30 nucleotide long constant linker elements. For the demultiplexing we allowed one mismatch in the barcode elements and when mapping with the constant elements we permitted 10 mismatches in both. To count the number of mismatches we ran ESGI to extract only the three barcode elements that serve as single-cell indices and count the edit operations during mapping. For correlations between the results of different tools we filtered the final count matrices for cells with >100 UMIs. The data are publicly available from GEO (accession GSE110823) and was downloaded from the Sequence Read Archive (SRA run SRR6750041).

## Results

### ESGI enables flexible pattern mapping

Most existing tools for barcoding-based single-cell sequencing data provide limited flexibility regarding barcode architecture, disallow insertions and deletions (indels) within barcode sequences, and lack support for mapping constant linker elements containing indels that might shift subsequent barcode positions. To address this, we developed ESGI to facilitate easy demultiplexing and counting even for complex barcoding technologies (Fig. 2). To showcase the usability of ESGI we selected six datasets from four novel technologies with custom barcoding schemes. We processed the datasets with ESGI and the tools that were used in the original publication of the respective datasets. All tools allow the mapping of a generic barcode pattern (Table 1). However, none of these pipelines supports indels or barcodes of variable length (staggers), because they all extract barcodes at predefined positions within the input read, whereas ESGI maps barcodes sequentially. Of note, alevin-fry does allow for variable-length barcodes for specific single-cell protocols such as inDropV2 and sci-RNA-seq3, but not for variable-length barcodes at any position within the read. Additionally, ESGI can process multiple barcode patterns simultaneously, enabling analysis of sequencing libraries that contain different modalities with distinct read structures or barcoding schemes with hierarchical organization and mutually-exclusive barcode sets (Supplementary information 1.1).

**Table 1:** Comparison of demultiplexing tools or workflows for single-cell data used for benchmarking (tool versions as of December 2025).

Name	generic BC pattern	insertions/ deletions	variable-length BCs	multiple patterns	modality	BC mapping	UMI correction	Reference
**alevin-fry** (salmon 1.10.0, alevin-fry 0.11.2)	$\textcolor{green}{\checkmark }$	$\textcolor{red}{\times }$	$\textcolor{red}{\times }$	$\textcolor{red}{\times }$	feature-barcodes/ RNA	position based	$\textcolor{green}{\checkmark }$	[15, 16]
**kallisto *∣ * bustools** (kITE) kallisto: 0.51.1, bustools: 0.45.1	$\textcolor{green}{\checkmark }$	$\textcolor{red}{\times }$	$\textcolor{red}{\times }$	$\textcolor{red}{\times }$	feature-barcodes/ RNA	position based	$\textcolor{red}{\times }$	[17, 18]
**zUMIs** (2.9.7e)	$\textcolor{green}{\checkmark }$	$\textcolor{red}{\times }$	$\textcolor{red}{\times }$	$\textcolor{red}{\times }$	RNA only	position based	$\textcolor{green}{\checkmark }$	[14]
**ESGI**	$\textcolor{green}{\checkmark }$	$\textcolor{green}{\checkmark }$	$\textcolor{green}{\checkmark }$	$\textcolor{green}{\checkmark }$	feature-barcodes/ RNA	sequentially	$\textcolor{green}{\checkmark }$	this work

In the following sections, we first apply ESGI to simulated combinatorial barcode data with known ground truth to quantify its accuracy in recovering indel-containing reads (Section ESGI  accurately  recovers  barcode  identities  from  simulated  indel-containing  reads). We then benchmark ESGI against established tools on six real datasets spanning combinatorial indexing, 10*× *-based single-barcode, and spatial barcoding technologies, demonstrating improved barcode recovery while maintaining high concordance with established pipelines (Table 2 and Section ESGI  improves  barcode  detection  and maintains  concordance  with  established  tools). We further show that ESGI achieves this flexibility with low memory usage and competitive runtime relative to other tools (Section ESGI  enables  efficient  barcode  mapping  with  low  memory  usage  and  fast  demultiplexing), and illustrate the detailed quality-control metrics ESGI provides to support debugging and optimization of novel barcoding designs (Section ESGI  provides  additional  information  on the  barcode  mapping  quality).

**Table 2:** Overview of datasets analyzed with ESGI.

Dataset	Modality	Barcoding scheme	Benchmarked against	Figure	Reference
Simulated data	Protein	2-round combinatorial indexing + feature BC	kITE, alevin	Fig. 3	—
SIGNAL-seq	Protein	3-round combinatorial indexing + feature BC	kITE	Fig. 4	[6]
SIGNAL-seq	RNA	3-round combinatorial indexing	zUMIs	Fig. 4	[6]
Phospho-seq	Protein	Single 10*× * cell barcode + feature BC	alevin	Fig. 4	[26]
Phospho-seq	RNA	Single 10*× * cell barcode	Cell Ranger	Fig. 4	[26]
xDBiT	Spatial	3 barcode elements (X, Y, sample)	xDBiT toolbox	Fig. 4	[1]
SPLiT-seq	RNA	3-round combinatorial indexing	STARsolo, alevin, kallisto/bustools	Fig. S4	[25]

### ESGI accurately recovers barcode identities from simulated indel-containing reads

To assess whether ESGI correctly identifies true barcodes carrying indels by mapping barcodes with Levenshtein distance, we simulated $1 \times 10^{6}$ reads with known ground-truth identities. We simulated a combinatorial barcoding scheme with feature barcodes. Feature barcodes are short nucleotide tags attached to antibodies that serve as a sequenceable readout for protein abundance. Feature barcodes have been widely adopted across multi-modal single-cell technologies, including CITE-seq for surface protein quantification via antibody-derived tags [5], REAP-seq [31], and intracellular epitope profiling approaches such as SIGNAL-seq [6] and Phospho-seq [26]. In our simulations, each read contained three barcodes: two combinatorial cell-identity barcodes and one feature barcode. To simulate sequencing errors, we introduced 1–3 errors per read by randomly selecting positions and the edit types applying substitutions, insertions, or deletions (see Section Simulation of reads from barcoded single-cell data). We then processed the reads with ESGI and two additional tools (kITE and alevin) that are both capable of processing combinatorial indexing data with feature barcodes. For kITE, direct read-level comparison with ESGI was possible, enabling quantification of true positive and false positive demultiplexed reads; alevin only reports the final single-cell feature matrix and does not report per-read barcode assignments before counting features for each cell and was therefore excluded from this analysis. We classified each recovered read as a true-positive if all three barcodes were recovered correctly. For the final count matrix evaluation in (Fig. 3c) we included alevin and the results of all three tools were compared against ground truth counts derived from simulated reads prior to error introduction.

**Figure 3.**
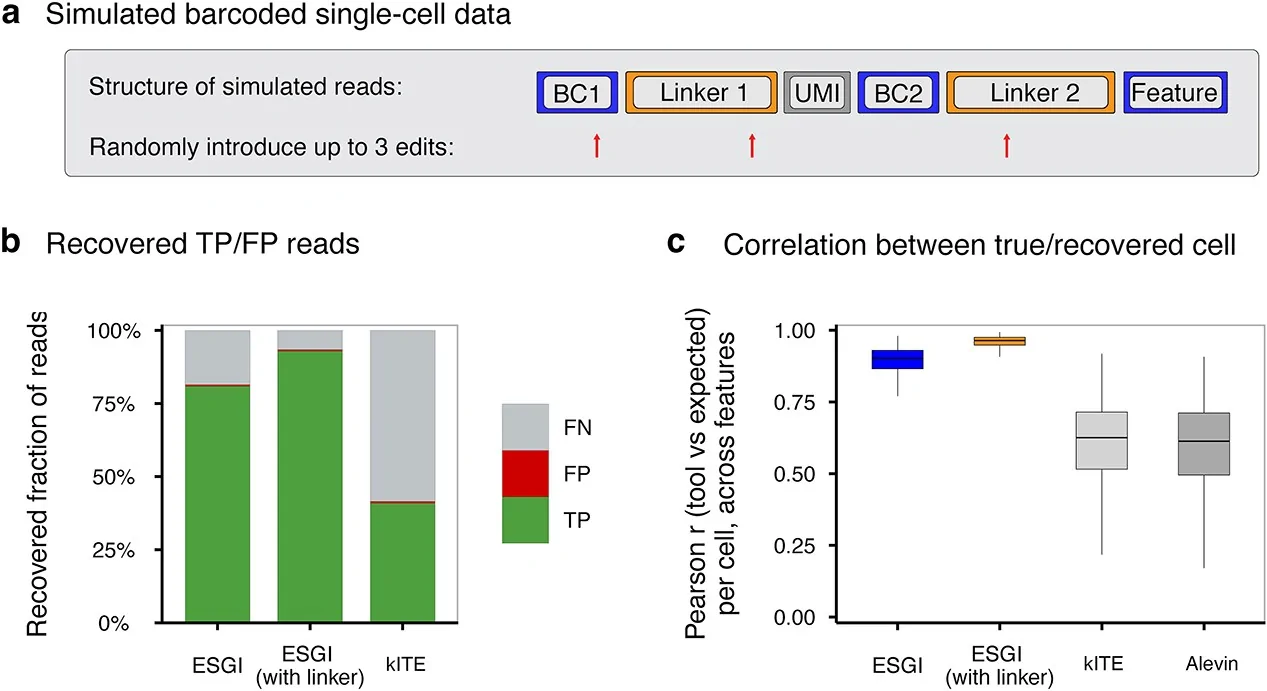
Recovery of simulated barcoded single-cell data. (a) We simulated 1 000 000 reads with a structure consisting of two cell barcodes (BC1 and BC2), a UMI, and a feature barcode to model single-cell identity and feature assignment. Sequencing errors were introduced at random positions with up to three edits per read, with equal probability of substitution, insertion, and deletion. (b) Read-level performance was assessed by classifying reads as true positives (correct recovery of both cell and feature barcodes) or false positives/false negatives based on barcode and feature assignment accuracy. (c) After UMI collapsing, we generated count matrices for each method and compared inferred counts against ground-truth counts. Each point corresponds to a single cell, and values represent the correlation between true and recovered feature counts within that cell.

First, we ran ESGI in a kITE-equivalent mode by disabling linker-aware mapping, such that linker sequences between barcodes were simply excised at fixed positions. In this mode, ESGI still considers indels within a barcode to shift all downstream barcode positions, but errors in the linker cannot be exploited to correct for such offsets. We counted the number of true and false positive reads as reads with correct or false barcode assignments, respectively. Compared with kITE, ESGI recovered a larger number of true positive reads, driven by its ability to rescue indel-containing reads that fixed-position methods like kITE cannot recover, while maintaining a low false positive rate (Fig. 3b). This demonstrates that ESGI’s increased sensitivity does not come at the expense of specificity, and the additional recovered reads are correct barcode assignments. Additionally, ESGI can leverage linker sequences to further improve read recovery. Insertions and deletions within linker regions shift the positions of downstream barcodes, causing fixed-position extraction to fail. Therefore, we ran ESGI by explicitly mapping linker sequences as well with up to three permitted errors, which further increased the true positive rate from 81% to 93%.

Finally, we assessed whether the higher true positive rate of ESGI translates into more accurate feature count matrices. For each recovered cell, we correlated tool-recovered feature counts with the ground-truth counts across all feature barcodes. ESGI recovered count profiles that correlate more strongly with the ground truth than either kITE or alevin, with linker-aware mapping further improving recovery (Fig. 3c). Together, these results demonstrate that ESGI’s Levenshtein-based barcode matching with linker-aware position correction recovers a larger fraction of true reads with high specificity, yielding count matrices that more faithfully reflect the underlying ground-truth expression profiles for the simulated data.

### ESGI improves barcode detection and maintains concordance with established tools

To assess ESGI’s performance on real sequencing data, we evaluated it on six datasets spanning both protein and RNA modalities across three technology types: combinatorial indexing (SIGNAL-seq and SPLiT-seq), a 10*× *-based single-barcode technology (Phospho-seq), and a spatial barcoding technology (xDBiT). For each dataset, we quantified the frequency and types of sequencing errors in barcode positions, compared the number of successfully demultiplexed reads against the tool used in the original study (reads that were successfully assigned single-cell barcodes and feature barcodes when present), and assessed count matrix concordance by computing per-cell Pearson correlations across features (Fig. 4 and Fig. S4).

**Figure 4.**
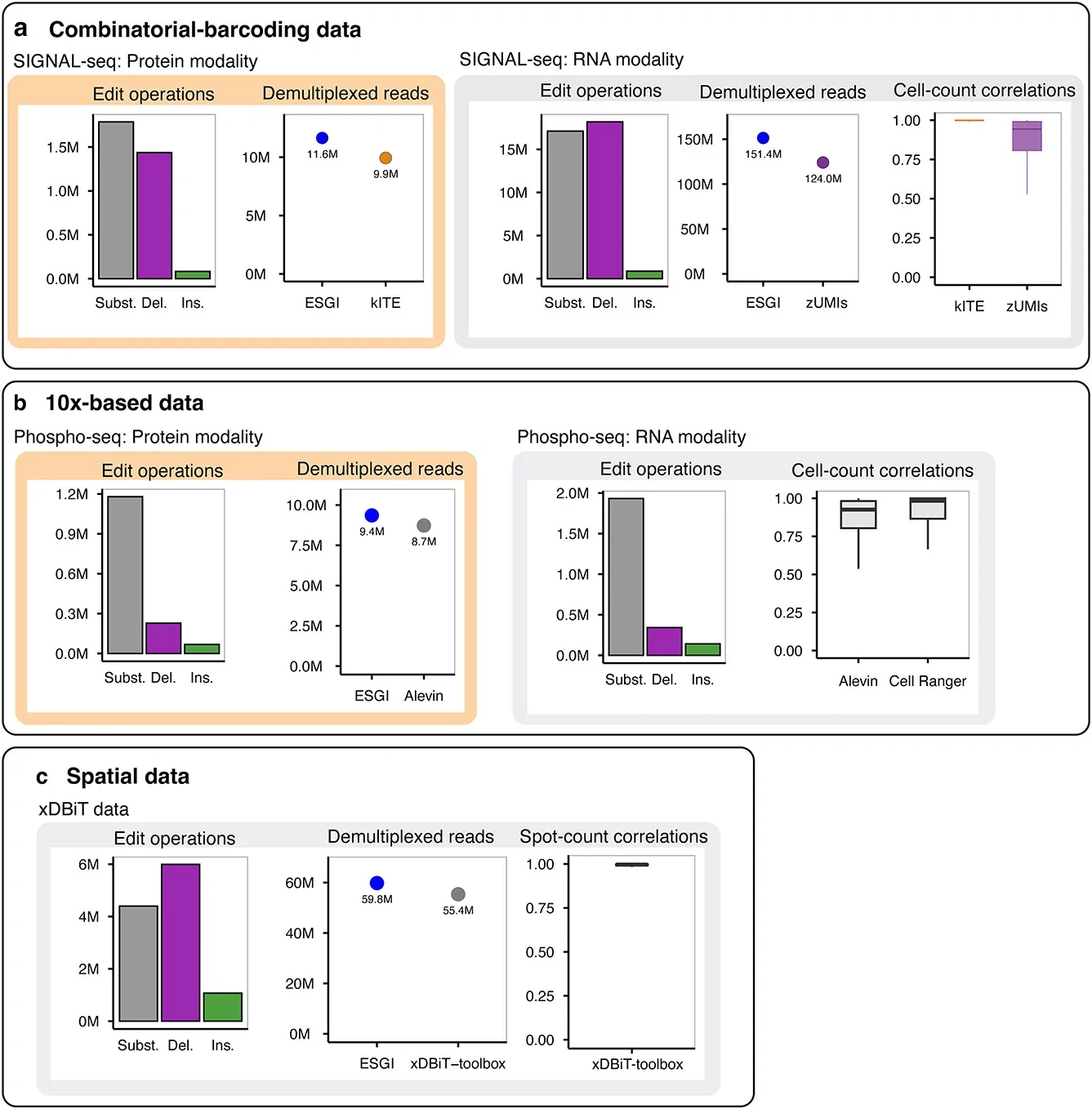
Overview of results across three technologies. Protein modalities are highlighted in yellow and RNA modalities in gray. For every technology we list the number of substitutions, deletions, and insertions measured with ESGI. Additionally, we show the number of demultiplexed reads for the various tools when applicable and boxplots of feature correlations. For the results of every tool we calculated Pearson correlations with the counts of ESGI per cell across all shared features. We evaluated ESGI on (a) data of the combinatorial-barcoding data of the SIGNAL-seq technology, (b) 10*× *-based data of the Phospho-seq technology and (c) the spatial barcoding technology xDBiT.

#### Combinatorial indexing: SIGNAL-seq and SPLiT-seq

Combinatorial barcoding schemes rely on multiple sequential barcode elements to encode single-cell identities, making them inherently sensitive to sequencing errors that occur early in the read. Insertions or deletions in an upstream barcode element shift the expected positions of all downstream barcodes, compromising their accurate assignment.

We applied ESGI to two datasets of SIGNAL-seq by Opzoomer *et al*., which profiles both RNA and protein expression using three rounds of combinatorial barcoding [6]. We analyzed paired protein and RNA measurements from HeLa spheroid cells treated with stimulants and inhibitors to induce diverse signaling states, followed by profiling of the transcriptome and a defined set of signaling proteins. We demultiplexed both modalities using ESGI and compared results with the original processing strategy, in which RNA reads were processed with zUMIs and protein-derived feature barcodes with kITE. In this comparison, ESGI was run without explicit linker mapping to match the fixed-position extraction used by kITE and zUMIs; results with linker-aware mapping are provided in the Supplementary Information (Fig. S6). For every successfully mapped barcode, ESGI records the number of substitutions, insertions, and deletions required to match a barcode, providing a detailed error profile for each mapped barcode sequence. Analysis of barcode error profiles revealed that indels are a prominent feature of combinatorial barcoding data. In the protein modality, the number of deletions was nearly as high as the number of substitutions, while in the RNA modality deletions exceeded substitutions, and the overall mismatch rate was higher (Fig. 4a). This is consistent with reported elevated deletion rates during oligonucleotide synthesis [23, 24] and underscores the need for indel-aware demultiplexing in combinatorial indexing experiments. Allowing for Levenshtein distance of one in each barcode element, ESGI recovered >10% additional reads compared to kITE and zUMIs for both modalities (Fig. 4a). This is further reflected in demultiplexing the same data with ESGI using Hamming versus Levenshtein distance, where permitting insertions and deletions via Levenshtein distance recovers >10% additional reads (Fig. S6).

To assess whether the increased read recovery translates to differences in downstream biological interpretation, we next compared count matrices generated by ESGI, kITE, and zUMIs on the SIGNAL-seq data. Count matrices generated by ESGI correlated highly with those of kITE and zUMIs, with a median Pearson correlation of 1.00 and 0.94, respectively (Fig. 4a). Direct comparison of count matrices across tools is inherently confounded by differences in quantification strategy independent of demultiplexing performance, including multi-mapping read handling, UMI collapsing algorithms (see Supplementary Information 1.3), and exon boundary definitions. To enable a more direct comparison, we first ran each tool with its own default parameters, and then re-ran ESGI using the UMI-collapsing settings that matched each tool’s default. We ran kITE and ESGI on the protein modality with zero mismatches and zUMIs and ESGI with one mismatch on the RNA modality (see Section Benchmarking ESGI on SIGNAL-seq data). Cells were retained when they met a lenient threshold of at least 100 counts per cell across both modalities. Under these conditions, ESGI retained 3.0 million protein counts in total compared with 2.6 million counts for kITE. ESGI also recovered more demultiplexed reads than zUMIs for the RNA modality (151 million versus 124 million). However, after assigning reads to genes and collapsing UMIs, zUMIs retained more counts per cell, with 6.5 million total counts and 33 000 genes detected, compared with 5.5 million counts and 25 000 genes for ESGI. This discrepancy despite ESGI’s higher read recovery must therefore stem from differences in gene-assignment logic: zUMIs relies on featureCounts [32], whereas ESGI relies directly on STAR’s GX/GN tags. The comparison at this stage therefore reflects featureCounts versus STAR’s built-in gene tagging, rather than a difference in the demultiplexing performance of ESGI and zUMIs. Despite recovering different numbers of demultiplexed reads, systematic processing differences of the tools can lead to divergence in the final count depth. Nevertheless, all tools yielded highly concordant biological outcomes, recovering the same major cell populations and cluster structure. Clustering and UMAP visualization recovered the same major cell populations for the protein and RNA modality for each tool (Fig. S5a), and cells from each cluster showed strong concordance between ESGI and kITE/zUMIs (Fig. S5b). Although total count depth varied between tools, per-cell counts were highly correlated across the full count matrix, indicating that the tools quantify individual cells consistently despite global differences in total read recovery (Fig. S5d,e). Taken together, these results demonstrate that while ESGI demultiplexes more reads, differences in downstream quantification strategies introduce variation in final count depth that is independent of demultiplexing performance, and all three tools converge on equivalent biological conclusions with highly concordant cell populations and cluster structure.

To further benchmark ESGI beyond the SIGNAL-seq data, we demultiplexed a dataset generated with SPLiT-seq and compared ESGI against STARsolo, alevin, and kallisto/bustools (Fig. S4). SPLiT-seq, developed by Rosenberg *et al*. [25], is a single-cell RNA-seq method that introduced combinatorial barcoding to label cellular origin through a split and pool approach. Here we used a dataset of 131 nuclei from the developing mouse brain. In SPLiT-seq, all three cell barcodes and the UMI are contained within a single read. This layout is compatible with STARsolo, which supports flexible barcode positioning but requires that all barcode information resides in one dedicated barcode read, and therefore cannot process protocols in which barcode segments are distributed across both paired-end reads [13]. The SPLiT-seq data contained ¿6million deletion errors alongside ¡8 million substitutions, and demultiplexing with ESGI recovered >10% additional reads relative to STARsolo. Alevin and kallisto/bustools do not expose barcode-level read assignment metrics that would allow an equivalent direct comparison. Per-cell count correlations were high across all tool comparisons, with ESGI and STARsolo showing the strongest mutual concordance, as did alevin and kallisto/bustools, consistent with their respective alignment strategies. ESGI and STARsolo both rely on STAR-based genomic alignment, while alevin and kallisto/bustools employ pseudoalignment, explaining the within-group agreement and modest divergence between groups.

#### Single-barcode technology: Phospho-seq

Phospho-seq, developed by Blair *et al*. [26], is a single-cell technology that quantifies intracellular proteins alongside chromatin accessibility and gene expression. We applied ESGI to a protein and RNA dataset of human iPSC-derived retinal organoids processed with the Phospho-seq-multi workflow, which jointly measures protein, RNA, and chromatin accessibility. In contrast to combinatorial indexing, the Phospho-seq technology encodes cell identity using a single-cell barcode, which eliminates the downstream position-shifting effect of indels in upstream barcode elements. Accordingly, the error profile of Phospho-seq data is dominated by substitutions, with indels occurring at substantially lower rates (Fig. 4b).

For the protein modality, 41% of reads matched the expected pattern exactly, and running ESGI with either Hamming or Levenshtein distance improved recovery only marginally (44% and 45%, respectively) (Fig. S6). For the RNA modality, ESGI mapped 75% of reads without any mismatch and recovered only an additional 1% with Hamming distance of one and 0.5% with Levenshtein distance (Fig. S6). Compared with alevin ESGI reported 8% more reads. Cell Ranger did not allow a comparison of recovered cell barcodes before gene annotation. Count matrices from ESGI, alevin, and Cell Ranger were highly concordant across both modalities (Fig. 4b).

#### Spatial barcoding: xDBiT

Finally, we applied ESGI to the spatial barcoding technology xDBiT, developed by Wirth *et al*. [1]. It measures transcriptomes of nine tissue sections in parallel at 50 *μ *m resolution and encodes the spatial position of captured molecules using two coordinate barcodes. The method was demonstrated on various tissues and we analyzed a cerebellum dataset. Similarly to the combinatorial indexing data, xDBiT data contain predominantly deletions (Fig. 4c). When comparing to the xDBiT toolbox, which also permits Levenshtein-distance matching during barcode lookup, there is an improvement of 9%. The xDBiT-toolbox still extracts barcodes from fixed read positions, meaning that indels in upstream sequence elements shift downstream barcode positions without correction. ESGI addresses this by assigning barcodes sequentially and updating the expected start position of each downstream element based on the outcome of the preceding match, thereby tolerating positional shifts caused by indels. The count matrices of ESGI and the xDBiT toolbox correlate highly. This might be attributed to the fact that xDBiT captures transcripts at the resolution of 50 *× * 50 *μ *m spots, each containing multiple cells, in contrast to the single-cell resolution of SIGNAL-seq and Phospho-seq. Averaging signal over many cells within a spot smooths out cell-to-cell variability which occurs when comparing correlations of single-cell counts.

### ESGI enables efficient barcode mapping with low memory usage and fast demultiplexing

To compare the runtime and memory usage of ESGI with that of kITE and zUMIs, we ran the tools five times on the protein or RNA modalities of the SIGNAL-seq dataset. kITE processed the 17 million reads of the protein data in under 1 min, while ESGI needed 8 min when it was run with a single thread and *∼ *2 min when run on 20 threads (Fig. 5a). For the RNA modality, ESGI processed the 183 million reads much faster than zUMIs, in *∼ *1 h compared with around 3 h for zUMIs (Fig. 5a). Of note, ESGI spent >79% of the time running STAR. The peak memory usage of ESGI was around 2 GB for the protein and below 40 GB for the RNA dataset (Fig. 5b). This was almost two times lower than kITE or zUMIs. For the RNA data, the majority of the memory usage was again attributed to STAR and the annotation of the demultiplexed reads (Fig. 5c). The computational demands of ESGI are driven almost entirely by STAR alignment, which accounted for >79% of total runtime and the majority of the memory consumption. In contrast, the barcode demultiplexing and count matrix generation steps are inherently lightweight and, in isolation, well within the reach of a standard laptop—underscoring that ESGI imposes no meaningful computational burden beyond that of the underlying aligner. Especially if the data contain only feature barcodes with no RNA modality that has to be mapped to a reference genome, ESGI can easily be run on a standard laptop (Table S1).

**Figure 5.**
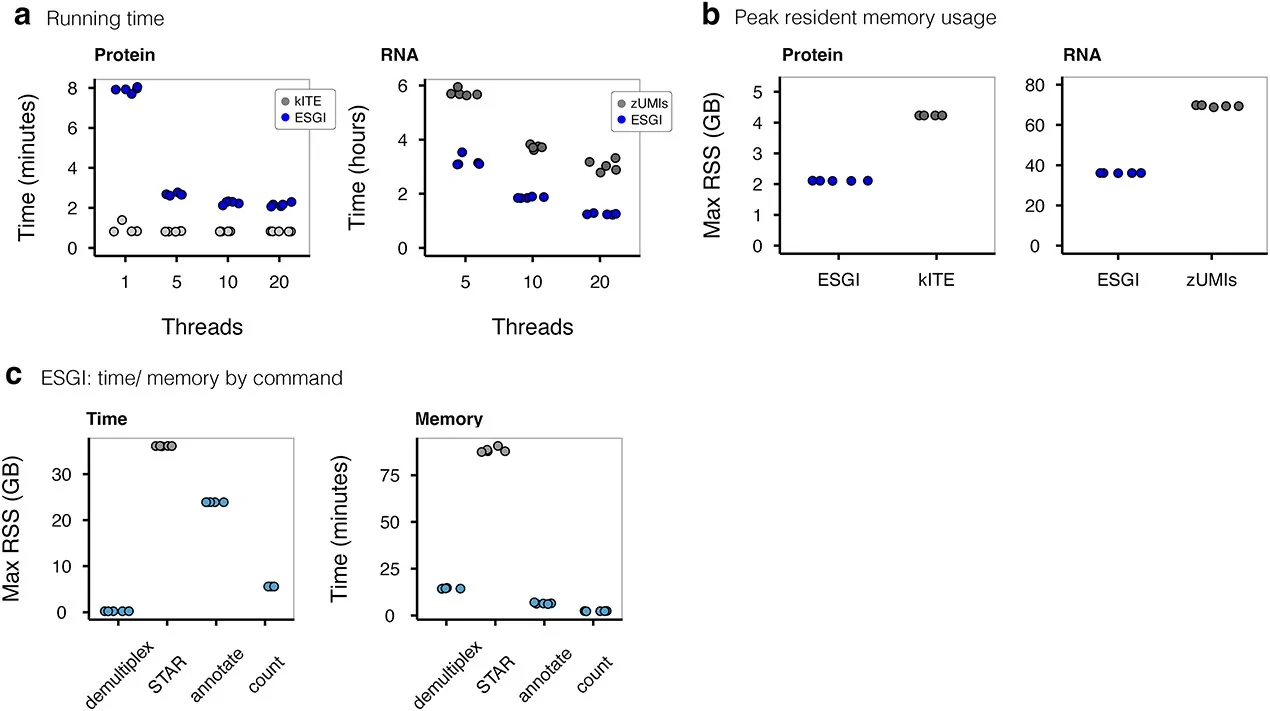
Runtime and memory usage of ESGI. (a) Running times of kITE, zUMIs, and ESGI for the protein and RNA modality demultiplexing. All tools were run five times. (b) Peak resident memory usage for kITE, zUMIs, and ESGI for the runs with 10 threads. (c) Running time spent in the individual commands of ESGI as well as peak resident memory usage per command for ESGI during processing of the RNA modality with 10 threads.

### ESGI provides additional information on the barcode mapping quality

To facilitate data quality control (QC), ESGI outputs position-resolved information about the mapping quality of the barcoding pattern. If a read does not map to the predefined pattern, ESGI reports the position in the pattern where mapping failed. To showcase this functionality, we evaluated the quality metrics of the protein modality of the SIGNAL-seq data. SIGNAL-seq uses feature barcodes as readouts of protein abundances in single cells and marks cells with a combination of three barcodes (Fig. 6a). Figure 6b shows at which position in the predefined input pattern the mapping failed when either allowing for zero mismatches or one mismatch in each of the barcode elements, and five in each of the constant elements. When allowing for zero mismatches in any element, the mapping fails most often in the first element of the forward read (AB) and the first element of the reverse read (BC3). However, when allowing for mismatches, mapping fails more evenly across the elements, with most errors occurring in the last position of the reverse read (BC1). Additionally, ESGI reports the number of mapped reads with and without mismatches for all positions in the pattern. Figure 6c shows the number of reads with zero or one mismatch in a barcode index for all three barcode positions (BC1, BC2, and BC3). The difference between reads with zero and one mismatch progressively decreases from BC3 to BC1. As BC3 is positioned at the start of the read—where sequencing quality is generally highest—this may facilitate more accurate mapping and reduce the impact of mismatches. Some barcodes were detected more frequently with one mismatch than with zero mismatches in BC1 and the variance in the number of reads is much higher in BC1. Figure 6d shows the number of substitutions, deletions and insertions for the various pattern positions in the reverse read. Overall, ESGI recorded a high number of deletions, consistent with previously reported high deletion rates in synthetic barcodes [23, 24]. Especially the first constant region shows a high deletion rate. If barcodes were not mapped sequentially these deletions could lead to a shift in the starting position for barcodes, which could then be missed.

**Figure 6.**
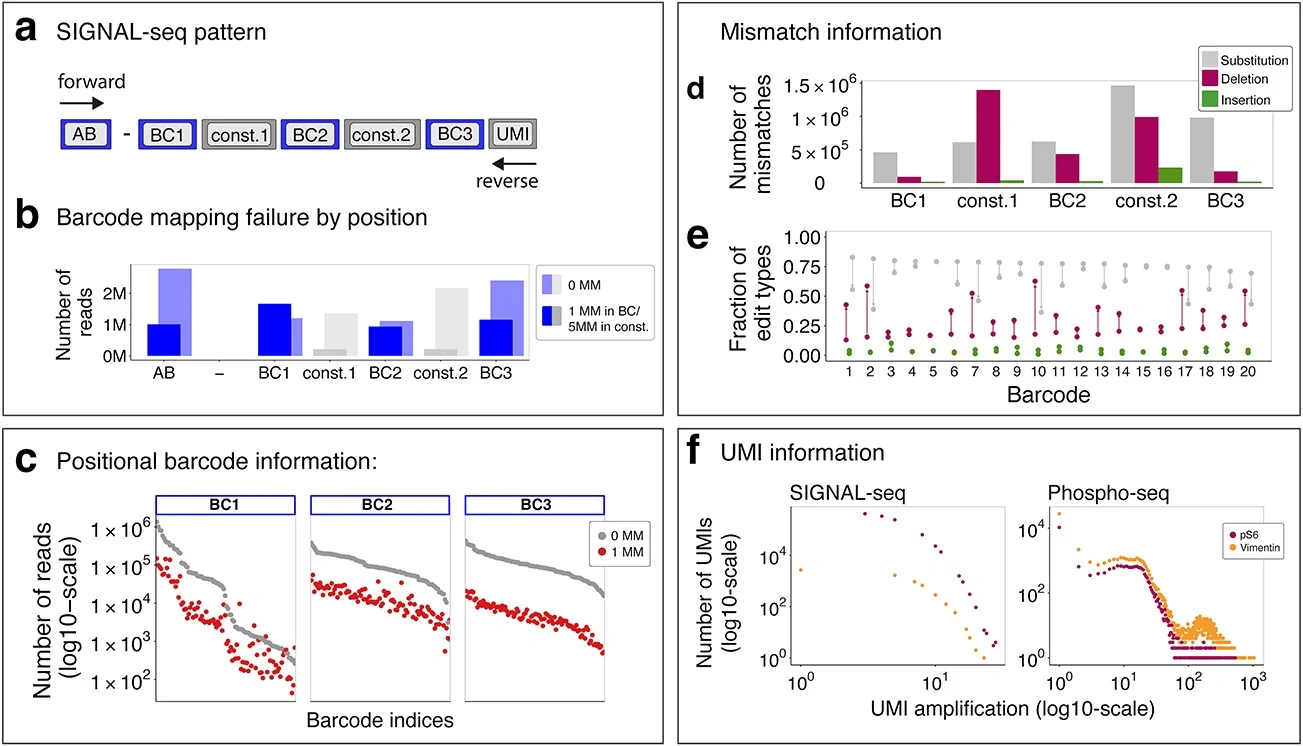
ESGI reports QC information. For the statistical information, we explicitly mapped the whole pattern of the SIGNAL-seq data: the antibody-tagged feature barcode (AB), three barcode elements encoding the single-cell identity (BC) and also the constant elements (const.1 and const.2). (a) The input pattern describing the barcoding layout of the SIGNAL-seq data. (b) The number of reads that failed at the different positions of the input pattern. ESGI was run allowing for zero mismatches (MM) or for one mismatch in the barcode elements and five mismatches in the constant elements. (c) Number of successfully demultiplexed reads for all barcodes in all three barcode positions. Every dot represents one barcode and barcodes are ordered in decreasing order by the number of reads that contain this barcode with zero mismatches. ESGI was run allowing for at most one mismatch in every barcode element. Gray depicts the number of reads that have zero mismatches in a barcode and red shows the number of reads with one mismatch in a barcode. (d) Number of edit-operations (substitution, insertion, and deletion) by position in the input pattern in the reads that were mapped successfully. (e) Fraction of reads with one substitution, insertion, or deletion for the first 20 barcodes in alphabetical order of the barcode elements BC2 and BC3. Both barcode elements share the same barcodes and arrows connect the barcodes of BC3 with the barcodes of BC2. (f) Distribution of UMI amplification for the two most abundant feature barcodes in the SIGNAL-seq and Phospho-seq data respectively.

Additionally, ESGI stores the number of different edits for all possible barcode indices in BC1, BC2, and BC3. Figure 6e shows the fraction of reads with different edits for the barcodes in the barcode elements BC2 and BC3. The fraction of deletions goes up drastically in BC2, while the fraction of insertions remains stable. Some barcodes (e.g. barcode 2 and 10) show a marked increase in the fraction of deletions between barcode elements BC3 and BC2, whereas others (e.g. barcode 3 and 4) display little to no change in deletion frequency. These barcode-specific differences in the distribution of insertions, deletions, and substitutions provide insight into how individual barcodes are differentially affected by editing events. Such information may help identify barcodes that are more susceptible to certain types of sequence alterations and can therefore inform future barcode design and optimization strategies. Finally, ESGI counts the number of reads for every observed UMI (before UMI correction). This makes it possible to plot the frequency of UMI numbers (Fig. 6f), which might serve as an additional metric to evaluate the sequencing depth or seperate true molecule reads from background noise that might be introduced during PCR [33, 34]. The distribution of UMI amplification differed between the SIGNAL-seq and Phospho-seq datasets (Fig. 6f). Phospho-seq showed a clear multimodal pattern, with a population of UMIs detected only once or twice, separated from distinct peaks of highly amplified UMIs, whereas SIGNAL-seq showed no such separation. Low-count UMIs are commonly attributed to spurious sequences arising from PCR or sequencing errors rather than genuinely captured molecules [35], whereas true molecules accumulate reads over successive amplification cycles [36]. A UMI-amplification threshold can therefore only be meaningfully defined when such a separation is present in the data. We envision that the QC metrics as shown in Fig. 6 provide a practical framework to improve or debug novel experimental technologies during their development phase and enable quantitative assessment of sequencing depth sufficiency.

## Discussion

Barcoding-based sequencing protocols are rapidly increasing in complexity, incorporating multiple molecular modalities, and increasingly elaborate barcoding schemes. In particular, combinatorial designs that rely on multiple rounds of barcoding can be affected by indels as insertions or deletions occurring early in the read can shift downstream barcode positions and degrade mapping accuracy. ESGI addresses these challenges by providing a flexible and generic demultiplexing framework that supports barcodes of variable length at arbitrary positions within single- or paired-end reads and enables indel-aware barcode matching. Moreover, ESGI allows multiple barcode patterns present within a single FASTQ file to be processed simultaneously, facilitating hierarchical barcode assignment and the joint processing of multimodal libraries. Together, ESGI provides a flexible framework for demultiplexing various barcoded short-read sequencing designs. ESGI makes it possible to quickly go from raw FASTQ files to the final single-cell feature matrices. It offers the user the possibility to set more specific parameters, such as the number of mismatches allowed in every individual barcode, and gain statistical information on the mapping output. These quality metrics can help to debug novel sequencing technologies, especially in the developmental phase.

Tools like kITE and alevin are specialized for processing genomic data, focus on extremely fast, and memory efficient mapping of transcripts to a reference genome and added features to handle existing barcoded single-cell protocols. In contrast, we developed ESGI with a focus on more flexibility in the demultiplexing step, making it especially suitable for novel experimental designs with complex barcoding schemes. ESGI is developed as a multi-threaded, RAM efficient tool. However, it relies on STAR for mapping of genomic sequences and therefore lacks the efficiency of software specialized for pseudoalignment, such as kallisto or alevin.

In this setup, STAR is effectively used in an exon-centric manner, as plain STAR gene assignment is restricted to exonic regions. Therefore, intronic read counting—required for RNA velocity analysis or nascent RNA quantification—is not natively supported and would require post-processing with dedicated tools, such as featureCounts [32]. Pipelines such as zUMIs or Cell Ranger integrate additional steps to explicitly support intronic counting (e.g. via featureCounts or internal intron-aware logic). Importantly, the *demultiplex* step of ESGI can be run independently, and be used to perform demultiplexing with flexible barcode pattern definitions and indel-aware barcode matching, after which the resulting RNA reads can be processed with downstream tools like featureCounts that support alignment to intronic as well as exonic regions.

Allowing for insertions and deletions (indels) may have limited impact in simple barcoding schemes with high sequence quality and saturated sequencing depth. However, explicit handling of indels becomes increasingly important for lower-quality data and for technologies employing complex barcoding strategies. The prevalence of indels is influenced by multiple factors, including barcode and sequence design, experimental protocol, and the number of amplification and barcoding steps. Especially in combinatorial barcoding approaches involving multiple rounds of barcode attachment, indels can shift the positions of downstream barcodes. ESGI explicitly addresses this challenge by implementing indel-aware barcode matching within user-defined flexible barcode patterns and can accurately identify barcodes even when their positions are altered. Although the additional reads recovered by ESGI did not reveal new biological structure in the datasets examined here, we anticipate that the benefit of higher read recovery will be more evident in more challenging settings, such as low-quality libraries or datasets with elevated barcode error rates, where discarded reads are less redundant with the remaining data.

A direct comparison of absolute counts between tools is inherently challenging, as differences in transcript mapping and UMI collapsing strategies can have a larger effect on the final count matrix than the demultiplexing step itself. Collapsing UMIs too aggressively merges distinct molecules and leads to undercounting, while insufficient collapsing retains sequencing errors as artificial counts and leads to overcounting. Tools differ in how they approach this problem: zUMIs, Cell Ranger, and alevin all permit mismatches when collapsing UMIs, while kallisto/bustools collapses UMIs without allowing for mismatches. Despite this diversity of strategies, a systematic benchmark of scRNA-seq preprocessing pipelines found that UMI collapsing strategy had less influence on downstream analytical results than normalization choices—suggesting that while absolute count numbers can differ between tools, the biological conclusions drawn from them are more robust [37]. This is also in line with our analysis of the SIGNAL-seq data, where we find the same biological clusters with highly correlated counts after processing the data with ESGI, kITE, and zUMIs (Fig. S5). Nevertheless, as we demonstrate in the Supplementary Material, the choice of collapsing strategy can alter counts and should be carefully considered when comparing count matrices across tools or when absolute quantification is required (Supplementary information 1.3). ESGI exposes both the mismatch threshold and an abundance-based collapsing criterion as user-defined parameters, allowing the collapsing behavior to be harmonized with that of other tools or tuned to the specific error profile of a given dataset.

While ESGI has been developed and validated exclusively on short-read sequencing data, long-read single-cell sequencing technologies represent an emerging and increasingly relevant application domain. Long-read single-cell approaches predominantly leverage established barcoding platforms such as 10*× * Chromium for library preparation, sequencing the same barcoded cDNA libraries on long-read instruments instead of Illumina, thereby retaining the same cell barcode and UMI structure while gaining access to full-length transcript information and isoform-level quantification [38]. However, barcode recovery from long-read data remains a fundamental challenge. Oxford Nanopore sequencing carries an error rate of 5%–15% compared with 1% for Illumina [39], and its error profile is dominated by deletions rather than substitutions. Current tools address this in different ways. Early approaches relied on hybrid sequencing, using matched Illumina short reads to generate a high-confidence barcode whitelist that guides assignment of the noisier Nanopore reads, improving barcode assignment rates but at the cost of requiring two independently constructed and sequenced libraries [40]. More recently, BLAZE enabled accurate cell barcode identification directly from Nanopore long-read data without requiring complementary short-read sequencing, though it remains restricted to 10*× * Chromium barcodes [41]. The deletion-dominated error profiles of long-read platforms are in principle well matched to ESGI’s Levenshtein distance-based barcode correction, which explicitly accounts for insertions and deletions, and ESGI’s flexible pattern framework could accommodate novel long-read barcoding designs that current tools cannot handle. However, the elevated overall error rates, longer read lengths, and chemistry-specific error distributions of long-read platforms would require dedicated benchmarking and potentially adaptation of alignment parameters before ESGI could be responsibly deployed in this context. We consider extension to long-read barcoded single-cell data an important direction for future development.

## Conclusion

ESGI supports processing of flexible barcode designs by enabling generic barcode patterns, indel-aware barcode processing and variable-length barcodes. These properties make ESGI particularly well suited for emerging sequencing technologies, for which standardized processing tools may not yet be available. We hope that ESGI becomes a useful tool for processing novel multiplexed single-cell sequencing technologies, without the need for tweaking existing tools or creating custom workflows. Additionally, the statistical information on the quality of mapping that ESGI provides, such as errors at various barcode positions, can provide valuable insight for refining highly multiplexed new technologies during their early development.

**Key Points**

- Efficient splitting of generic indices (ESGI) is a flexible framework for demultiplexing and processing single-cell sequencing data with arbitrary barcode architectures.
- The method supports variable-length barcodes and indel-aware barcode matching using Levenshtein distance, overcoming limitations of fixed-position and substitution-only pipelines.
- ESGI operates directly on raw FASTQ files and integrates barcode demultiplexing, optional genome alignment, UMI collapsing, and feature quantification.
- The framework can process diverse single-cell technologies, including combinatorial indexing, multimodal assays, and spatial barcoding experiments.

## Supplementary Material

supplements_bbag631

## Data Availability

ESGI is written in C++ and released under GNU General Public License (GPL) v3.0. ESGI can be obtained either as ready-to-use precompiled binaries or by compiling the source code, both of which are freely available at https://github.com/tstohn/ESGI. A documentation with examples how to use ESGI is available at: https://tstohn.github.io/ESGI.documentation. The code for the analyses of this paper is available at https://github.com/tstohn/Analysis-ESGI and the source code used in this study is available at Zenodo: https://doi.org/10.5281/zenodo.18887035. The datasets analyzed in this study are publicly available in the Gene Expression Omnibus (GEO) under accession numbers GSE256405, GSE285561, GSE207843, and GSE110823; sequencing reads were obtained from the Sequence Read Archive (SRA) under the corresponding run accessions reported in the Materials and methods.
