# Supplementary material for: ESGI: efficient splitting of generic indices in single-cell sequencing data
Source: Brief Bioinform. 2026 Nov 15;27(6):bbag631. doi: 10.1093/bib/bbag631 (PMC13770249; doi:10.1093/bib/bbag631)
Supplement: Supplementary file 1 [file supplements_bbag631.pdf]

Supplementary Information to: ESGI: Efficient splitting of generic indices in single-cell sequencing data

1 SUPPLEMENTARY NOTES

1.1 Multi-pattern mapping

Hierarchical and dependency-based barcode patterns pose a particular challenge for conventional demultiplexing tools, as they violate the assumption of fixed-length, positionally independent barcode elements. In such designs, the identity and length of barcode segments may depend on preceding elements, leading to variable-length and structurally interdependent barcode patterns. One example is scID-seq, a well-based technology to measure single-cell protein abundances [1]. The technology employs a stagger-based design in which the set of valid barcodes within a barcode element is constrained by the length of an upstream stagger element (Fig. S1a). ESGI's demultiplexing module enables explicit modeling of inter-element dependencies by representing each valid stagger–barcode combination as an independent pattern (Fig. S1b). For each read, ESGI systematically evaluates all specified patterns and assigns barcodes when a unique pattern match is identified.

1.2 Barcode mapping procedure in `demultiplex`

`demultiplex` identifies barcode patterns in sequencing reads by matching the user-defined pattern to input sequences (e.g., FASTQ reads). An overview of the mapping strategy is shown in Supplementary Fig. S2.

1.2.1 Hash-based barcode lookup

To efficiently resolve barcode identities at positions where multiple barcode candidates are possible, `demultiplex` uses as a first step precalculated hash tables (Supplementary Fig. S2b).

It performs direct lookups using a precomputed substitution map that links every possible sequence (including sequences containing a single substitution) to the corresponding original barcode. Per default a second map including all sequences with a single insertion or deletion (Levenshtein distance of one) is used. This indel-aware map can be disabled to reduce memory usage when only Hamming-distance matching is required. Only uniquely mapped reads are retained.

1.2.2 Positional *k*-mer filtering

To keep memory low possible sequences with more than two mismatches are not precalculated. When more than one mismatch is allowed, or when indel-aware matching is requested without using the precomputed Levenshtein map, `demultiplex` applies a positional *k*-mer filtering strategy (Supplementary Fig. S2b).

For all candidate barcodes, the algorithm generates every possible *k*-mer and stores it together with its positional index as a hash key. Each key maps to the barcode(s) in which the corresponding positional *k*-mer occurs. During filtering, positional *k*-mers are generated from the observed sequence and matched against this map to retain only compatible barcode candidates. A barcode is retained if it shares at least a minimum number of positional *k*-mers with the observed sequence. The minimum number of required matches is defined as:

$$N_{\text{min}} = (L_{\text{bc}} - L_k + 1) - m \cdot L_k,$$

where L_{bc} denotes the barcode length, L_k the *k*-mer length, and m the maximum number of allowed mismatches.

To account for indels, positional indices of *k*-mers are allowed to shift within a window that is of length of the number of permitted mismatches (positional *k*-mers can occur at the expected position +/- mismatches).

1.2.3 Final alignment

After filtering, remaining candidate barcodes are aligned to the extracted read segment. If a unique best match is identified, the read is assigned accordingly. Only uniquely mapped reads are retained. Barcodes are mapped with a semi-global alignment strategy (Fig. S3).

1.3 UMI collapsing

Various UMI collapsing strategies have been proposed and how UMIs are collapsed has a strong influence on the number of counts that are detected (Fig. S7). Collapsing UMIs wrongfully leads to an under or overestimation of counts, especially for features that are generally highly abundant. Many pipelines like zUMIs [2], Cell Ranger [3] or alevin [4] consider mismatches when collapsing UMIs, while other tools like kallisto and bustools [5] collapses UMIs without allowing for mismatches, which can lead to over-collapsing of UMIs. Many methods apply graph-based approaches merging similar UMIs like in alevin. The tool dropEst provides a Bayesian approach to demultiplex UMIs [6]. During UMI collapsing some tools additionally consider the base quality or abundance of reads to inform the collapsing of UMIs. Alevin for example collapses the less abundant UMI only into the more abundant one if its counts are less or equal to half the counts of the more abundant UMI (equivalent to the 50% collapsing rule in (Fig. S7)). A benchmark of various processing strategies for scRNA-seq data with UMI counts found that various UMI preprocessing methods had less of an impact on the results of the analysis than for example normalization strategies [7]. Nevertheless, for optimal results the collapsing of UMIs should be handled with care as counts can differ a lot depending on the chosen UMI collapsing strategy (Fig. S7).

To enable user-defined UMI collapsing, we provide two parameters: mismatches (default = 1), which specifies the maximum allowed Hamming distance between UMIs, and umiAbundanceThreshold (default = 0.2), which defines the relative abundance threshold for error correction. UMIs within the specified Hamming distance are eligible for collapsing if the less abundant UMI has fewer counts than the specified fraction (between 0 and 1) of the more abundant UMI. By default, UMIs differing by one mismatch are collapsed when the lower-abundance UMI has less than 20% of the counts of the higher-abundance UMI.

2 SUPPLEMENTARY FIGURES

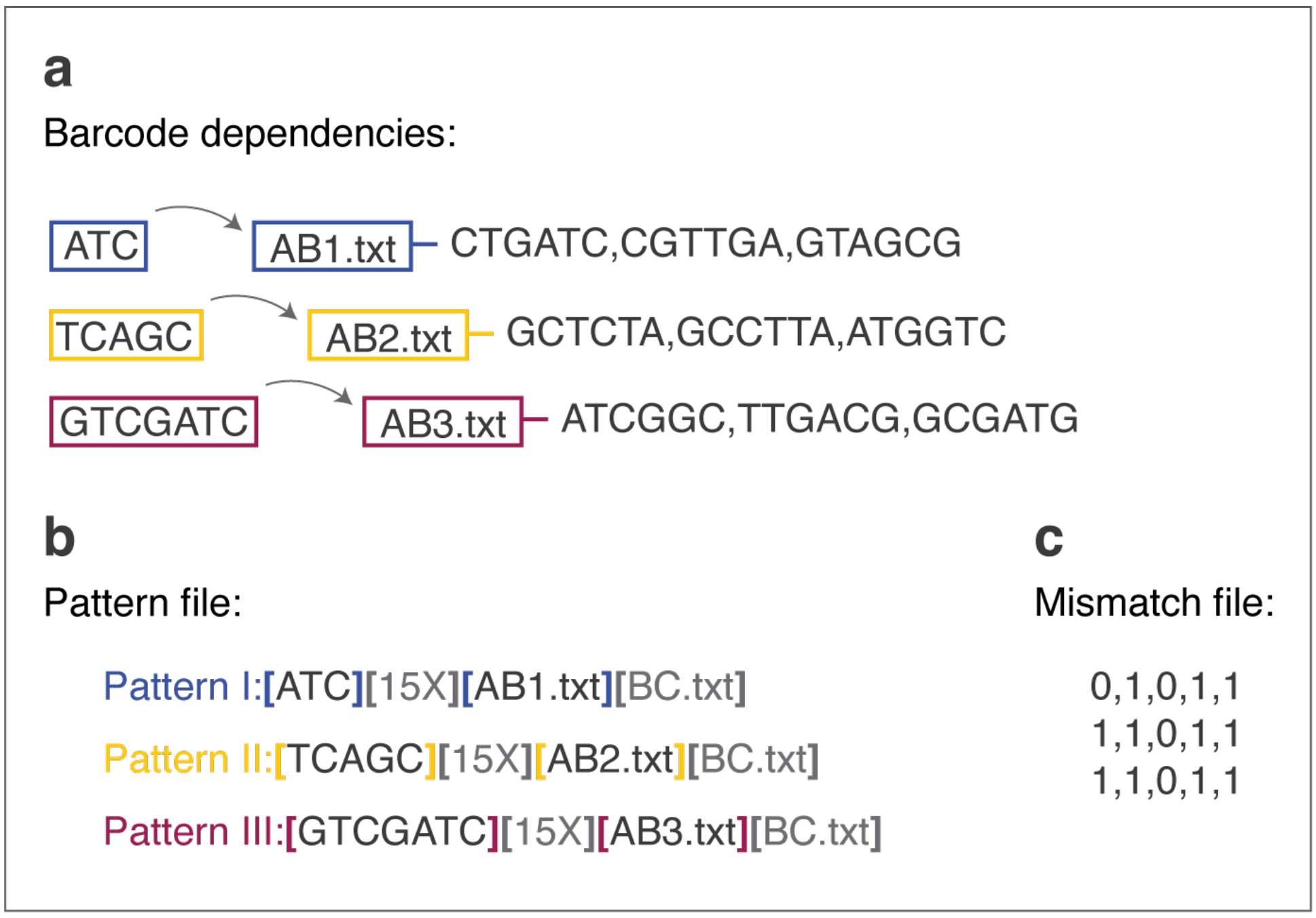

Figure S1: Demultiplexing multipattern designs with barcode dependencies. A.) To expand the barcode design space, dependencies can be introduced between discrete barcode elements within a pattern. In this example, the identity encoded by a barcode element is constrained by the length of a constant stagger element (ATC, TCAGC, GTCGATC). These staggers vary in length, and each is associated with a specific substring of potential sequences for the barcode element. B.) Each unique stagger-barcode combination is assigned a distinct pattern name and set of pattern elements. Each element is enclosed in brackets and can contain a constant sequence (stagger), random sequence (15X), or barcode (.txt). Pattern-specific files (AB.txt) encode the feature identity, while shared files (BC.txt) encode the single-cell identity. C.) For each pattern, the maximum allowed mismatches per element are defined as a comma-separated list of integers.

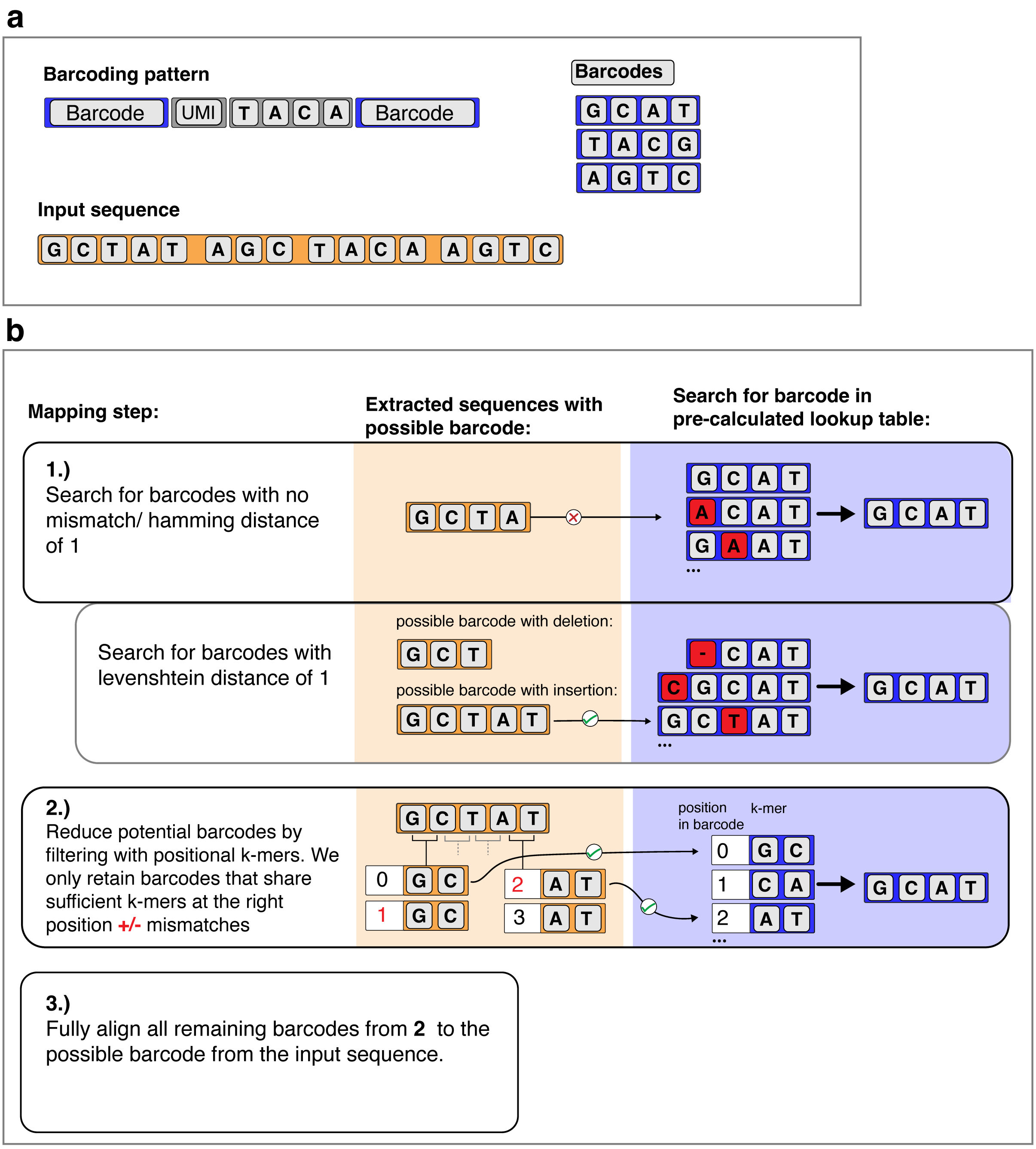

Figure S2: Overview of the barcode mapping workflow. (a) Example barcode pattern consisting of multiple barcode elements (blue), a UMI and a constant sequence (grey) that are mapped to input reads (yellow). (b) Three-stage barcode identification strategy combining hash-based direct lookup and positional *k*-mer filtering, followed by final alignment.

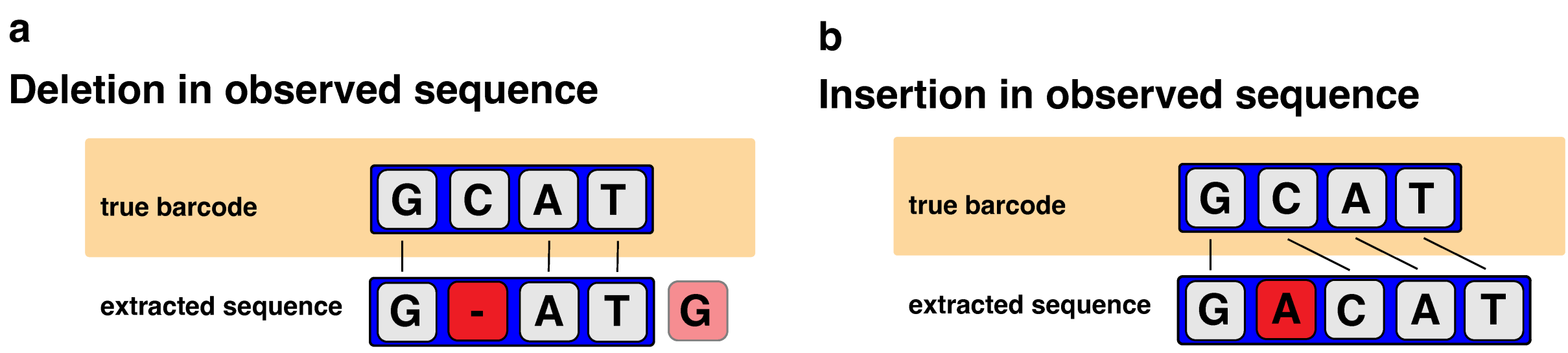

Figure S3: Semi-global alignment for barcode demultiplexing. Let L denote the expected barcode length and k the maximum number of allowed mismatches, including insertions and deletions. `demultiplex` extracts a sequence of length $L + k$ from a FASTQ read and aligns it to the expected barcodes using semi-global alignments. In this example, $L = 4$ and $k = 1$, such that a sequence of length 5 is extracted. (a) When the number of deletions exceeds the number of insertions, the observed sequence contains additional bases that are not part of the mapped barcode (blue background). The semi-global alignment allows unpenalized overhangs at the end of the mapped barcode in the extracted sequence (light red), while deletions occurring within the barcode are penalized (dark red). (b) When the number of insertions does not exceed k, the observed sequence has the correct effective length, enabling correct barcode identification within the whole length of the extracted sequence.

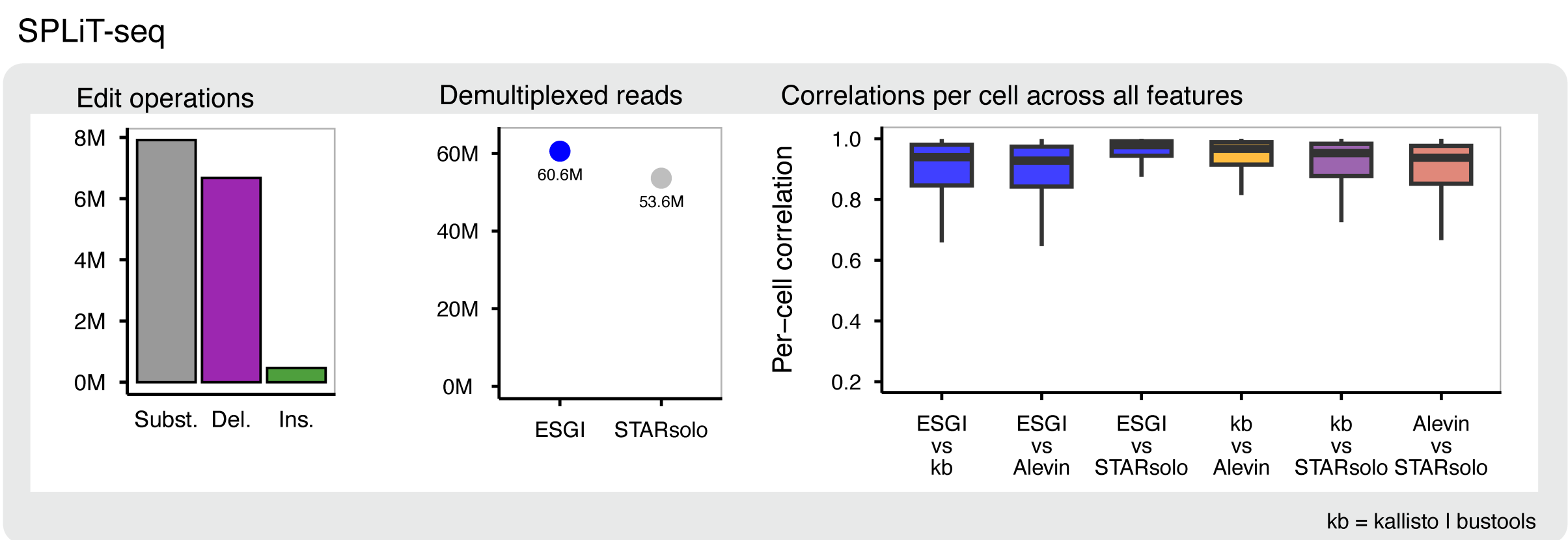

Figure S4: Analysis of SPLiT-seq data. Similar to the other datasets we ran ESGI on SPLiT-seq data to detect the number of errors in the barcodes of the SPLiT-seq data. Additionally, we compared the number of demultiplexed reads between ESGI and STARsolo. Finally, we calculated per feature correlations across cells between the count matrices for all pairs of tools.

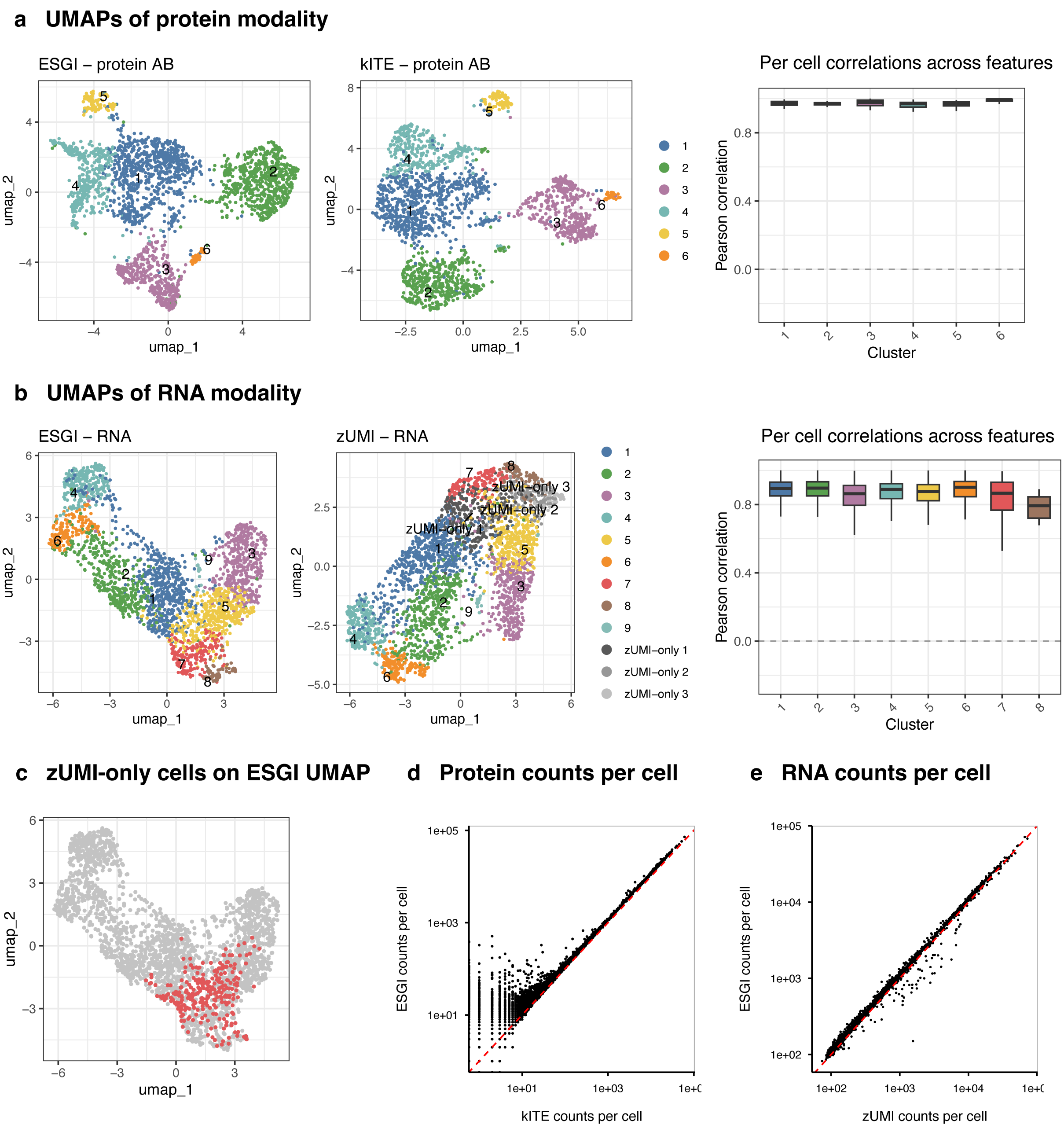

Figure S5: ESGI shows comparable biological results as tools used for the SIGNAL-seq data.. (a) UMAPs of the protein count matrices generated with ESGI and kITE. Matched clusters are highlighted in the same color (cf. Methods). The Pearson correlation per cluster show correlations for all shared cells across proteins. (b) UMAPs of the RNA count matrices generated with ESGI and zUMI, with matched clusters highlighted in the same color. Applying a minimum threshold of 100 UMIs yielded 2,365 cells for ESGI and 2,813 cells for zUMI. Notably, the original study employed a stricter multimodal filter combining protein and RNA data, retaining 1,143 cells. Pearson correlation coefficients per cluster demonstrate gene expression concordance across all shared cells. (c) At the same clustering granularity, zUMI detects three additional clusters (478 cells total). ESGI independently detects 269 of these cells, which are highlighted in red on the ESGI UMAP. (d) Scatterplot of the counts per cell that were generated with kITE and ESGI and (e) scatterplot of the gene counts for zUMI and ESGI.

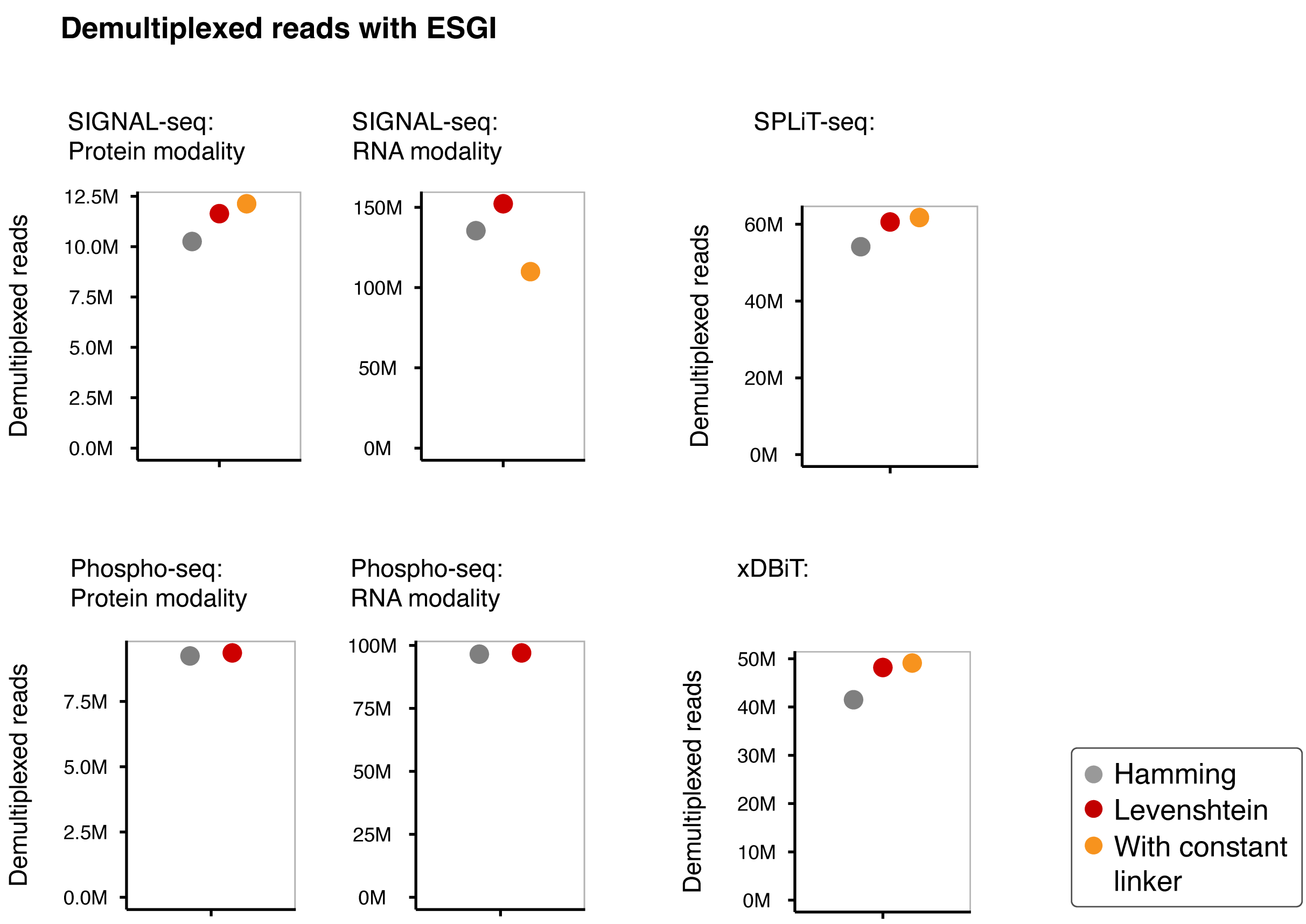

Figure S6: Number of demultiplexed reads with ESGI. We demultiplexed all datasets with ESGI allowing 1 edit within every barcode. We ran ESGI allowing for substitutions (Hamming distance in grey) or additional indels in the single-cell and feature barcodes (Levenshtein in red). For all datatsets that contained several barcodes that are connected through linker sequences we also ran ESGI with explicitly mapping the linker sequence with indels and allowing up to 10 edits (yellow). Explicitely mapping the linker sequence increase the number of demultiplexed reads for most technologies except the RNA modality of the SIGNAL-seq data. Notably, this dataset contained base-positions with Phred scores below 20 (Fig. S8)

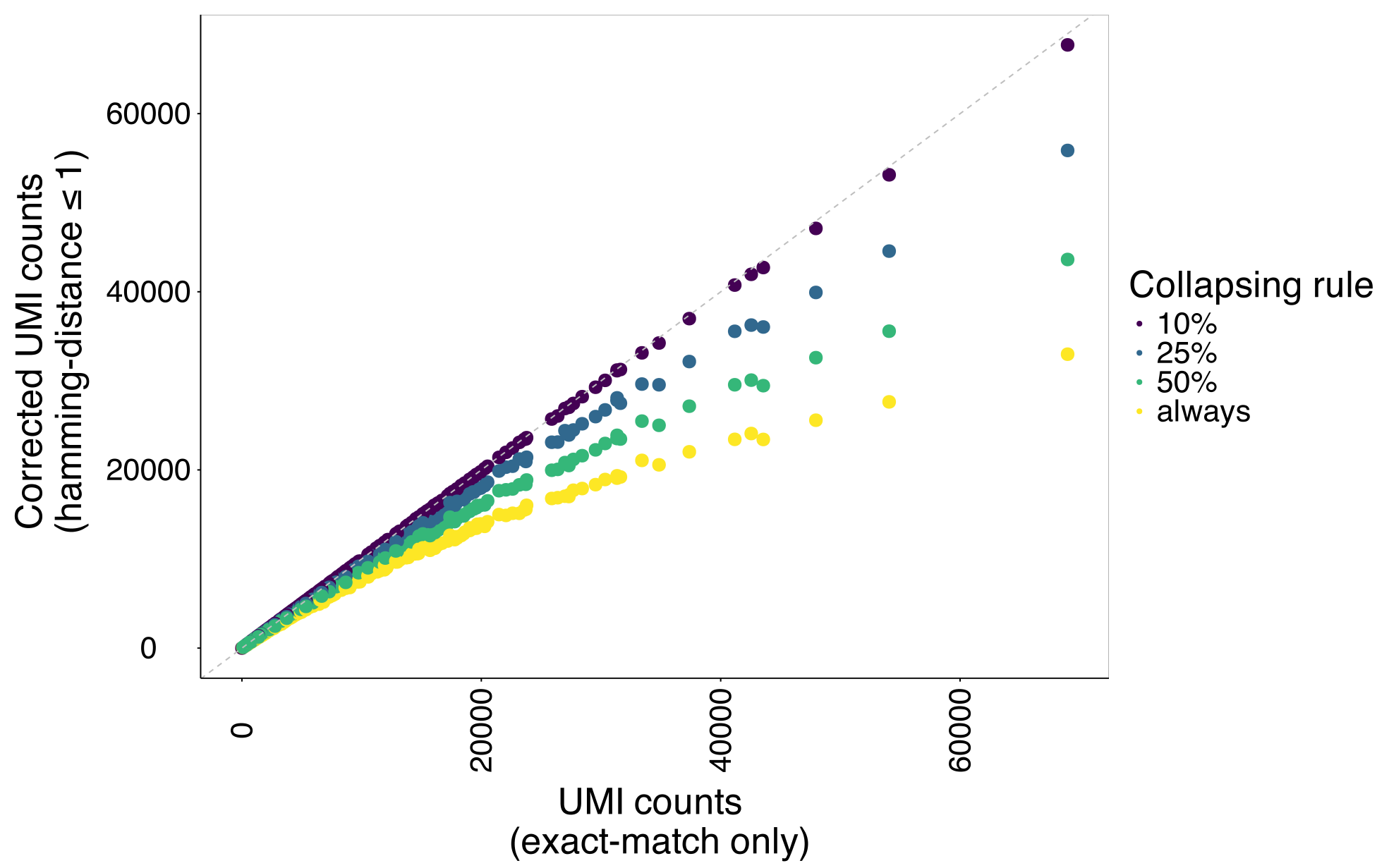

Figure S7: Collapsing of UMIs with ESGI (`count`-tool) for the protein modality of the SIGNAL-seq dataset. For every feature in every detected cell collapsed UMI counts without error correction (0 mismatches) are plotted against UMI counts that were corrected for mismatches (Hamming distance $<= 1$). Additionally, UMIs were only collapsed if the less abundant UMI was less than 10%, 25%, 50% of the more abundant UMI by setting the `umiAbundanceThreshold` parameter accordingly, or it was collapsed regardless of the UMI abundance (always).

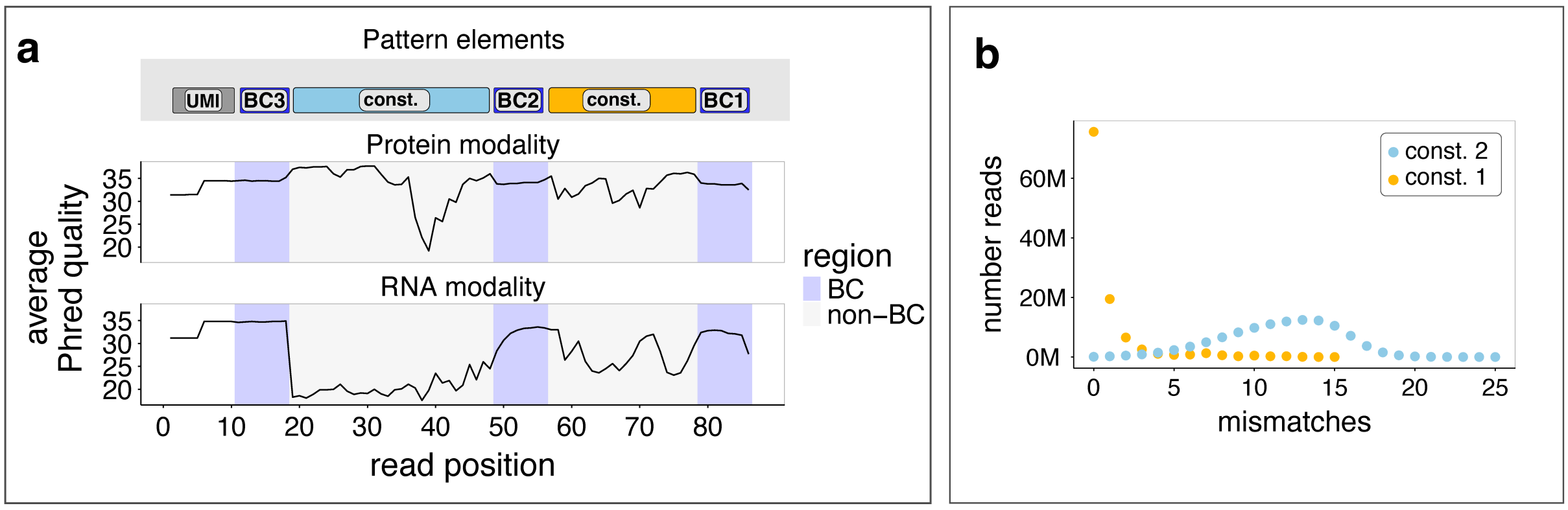

Figure S8: Average base quality of the reverse read containing the three single cell barcodes of the SIGNAL-seq data. (a) The average Phred score per position for the bases in the FASTQ file of the protein and the RNA modality. Because ESGI allows the mismatch tolerance to be defined separately for each pattern element, users can adapt the permitted number of mismatches according to the observed sequencing quality (e.g., Phred score distributions) of individual barcode or constant regions. (b) The number of mismatches in the two constant elements in the pattern for the RNA modality. We ran ESGI allowing for 15 mismatches in the first constant region and 25 mismatches in the second constant region and counted the number of mismatches (up to 15 and 25) for all reads that could be successfully mapped to the pattern.

Table S1: Recommended computational requirements for running ESGI, based on runtime and peak memory usage observed for the SIGNAL-seq dataset (Fig. 3)).

Task	Dataset size	Peak memory	Approx. running time
Feature-barcode only	17 M reads	$\sim$2 GB	$<$2 min (20 threads)<br>$\sim$8 min (1 thread)
RNA modality<br>(with STAR mapping)	183 M reads	$<$40 GB	$\sim$1 h (10 threads)

SUPPLEMENTARY REFERENCES

1. Eijl, R. A. v., Buggenum, J. A. v., Tanis, S. E., Hendriks, J. & Mulder, K. W. Single-Cell ID-seq Reveals Dynamic BMP Pathway Activation Upstream of the MAF/MAFB-Program in Epidermal Differentiation. *iScience* **9,** 412–422 (2018).
2. Parekh, S., Ziegenhain, C., Vieth, B., Enard, W. & Hellmann, I. zUMIs - A fast and flexible pipeline to process RNA sequencing data with UMIs. *GigaScience* **7,** giy059 (2018).
3. Zheng, G. X. Y. *et al.* Massively parallel digital transcriptional profiling of single cells. *Nature Communications* **8,** 14049 (2017).
4. Srivastava, A., Malik, L., Smith, T., Sudbery, I. & Patro, R. Alevin efficiently estimates accurate gene abundances from dscRNA-seq data. *Genome biology* **20,** 65 (2019).
5. Melsted, P. *et al.* Modular, efficient and constant-memory single-cell RNA-seq preprocessing. *Nature Biotechnology* **39,** 813–818 (2021).
6. Petukhov, V. *et al.* dropEst: pipeline for accurate estimation of molecular counts in droplet-based single-cell RNA-seq experiments. *Genome Biology* **19,** 78 (2018).
7. You, Y. *et al.* Benchmarking UMI-based single-cell RNA-seq preprocessing workflows. *Genome Biology* **22,** 339 (2021).
